# BOLL‐Containing Aggregates Mediate the Translational Regulation During Human Oogenesis

**DOI:** 10.1111/cpr.70181

**Published:** 2026-02-25

**Authors:** Ying Li, Lingya Mao, Boyuan Liang, Longxin Xie, Wenpei Xiang, Kehkooi Kee

**Affiliations:** ^1^ The State Key Laboratory for Complex, Severe, and Rare Diseases; SXMU‐Tsinghua Collaborative Innovation Center for Frontier Medicine; School of Basic Medical Sciences, Tsinghua Medicine Tsinghua University Beijing China; ^2^ Institute of Reproductive Health, Tongji Medical College Huazhong University of Science and Technology Wuhan Hubei China

**Keywords:** human BOLL protein, oogenesis, protein aggregate, translational regulation

## Abstract

Human oocyte meiosis utilises a specialised translational control strategy to coordinate meiotic progression, mediated through dynamic regulation of mRNA stores. While germ cell‐specific RNA‐binding proteins (RBPs) are known to orchestrate this post‐transcriptional programme, the mechanistic basis of RBP‐mediated cell fate specification remains elusive. Here, we demonstrate that BOLL, a Deleted in Azoospermia (DAZ) family protein, forms protein aggregates during meiotic prophase to drive translational reprogramming in human oogenesis. We determined that BOLL enhances the translation efficiency of cell cycle regulators, as demonstrated by integrative translatome‐transcriptome analysis combined with RNA immunoprecipitation sequencing. We also revealed the functional interaction network of BOLL with core translation machinery components through its conserved DAZ‐containing domain. Crucially, we identified SDS‐resistant protein aggregates as a structural signature of BOLL in human oocyte‐like cells, demonstrated by semi‐denaturing electrophoretic analysis. Using human foetal ovarian tissues and an hESC‐derived oogenesis model, we delineate a paradigm wherein BOLL‐containing aggregates exert spatiotemporal control over cell cycle genes during meiosis prophase. These findings reveal that protein aggregates of gametogenesis‐specific RBPs constitute an evolutionarily conserved mechanism in mammalian reproductive regulation.

## Graphical abstract

This work elucidates that BOLL‐containing aggregate‐mediated translational control is essential for human oogenesis. These aggregates recruit PABPC1 and FXR1 to activate the translation of U‐rich mRNAs encoding cell cycle proteins, thereby ensuring successful meiotic progression.

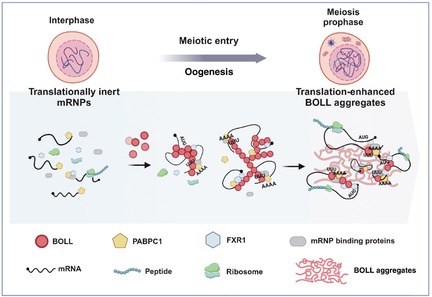

## 1 Introduction

Meiotic initiation in oogonia marks the critical transition towards oocyte development, a process fundamentally dependent on post‐transcriptional regulation as germ cells undergo transcriptional silencing during meiotic entry [1, 2, 3]. Germ cell‐specific RBPs are known to orchestrate this post‐transcriptional programme through dynamic control of pre‐store mRNA stability, localization and translation [4, 5, 6, 7]. Emerging evidence suggests that certain RBPs execute their regulatory functions through phase separation‐mediated formation of membrane‐less structures [8]. While phase separation of RBPs has emerged as a conserved regulatory mechanism across species, the biophysical principles governing protein aggregation and their functional consequences in mammalian oogenesis remain poorly defined.

Evolutionary insights from yeast reveal that a conserved RBP, Rim4, regulates meiotic progression through amyloid‐like aggregation, where its low‐complexity prion domain mediates translational repression of target mRNAs [2, 9, 10]. Notably, the human DAZ protein family, comprising Y‐linked DAZ genes and autosomal homologues BOLL/DAZL, shares conserved RNA recognition motifs and aggregation‐prone sequences resembling yeast prion‐like domains [11, 12, 13, 14, 15]. Although murine studies demonstrate testicular aggregate formation by DAZL and BOLL [10], their capacity to undergo protein aggregation during mammalian oogenesis remains unexplored. The evolutionary conservation of BOLL prion‐like domains across metazoans [11], coupled with its ancestral position in the DAZ family phylogeny, suggests that BOLL‐mediated protein aggregation may represent an ancient mechanism for meiotic cell cycle control. Resolving the mechanistic relationship between BOLL aggregation states and cell cycle‐related mRNA regulation may therefore unveil fundamental principles underlying human fertility disorders.

Studies in model organisms have established the essential function of boule in meiosis prophase I. In *Schmidtea mediterranea* , knockdown of the boule ortholog boule1 results in oocyte depletion [16], while in *Caenorhabditis elegans* , boule mutation causes meiotic arrest in oocytes [17]. However, the regulatory mechanisms governing boule during meiosis remain poorly defined. Although enhanced cross‐linking and immunoprecipitation (eCLIP) analyses in male mice have identified BOLL‐bound mRNAs critical for spermatogenesis [18], its oogenesis‐specific targets and stage‐specific regulatory logic in human female germ cells remain uncharacterized. This knowledge gap is largely due to the scarcity of clinical oocyte samples at meiotic prophase I, a stage of specific BOLL expression [1]. To address this limitation, human embryonic stem cell (hESC)‐derived in vitro oogenesis models have been utilised to investigate the mechanisms of BOLL in female germ cell development. Recent studies using human induced pluripotent stem cells (iPSCs) demonstrate that BOLL overexpression promotes meiotic entry and differentiation [19], supporting its function in driving meiotic fate commitment in pluripotent cells.

In this study, we constructed a human oocyte‐like cells (OLCs) system by combining BMP4 treatment and BOLL overexpression. Using integrated multi‐omics profiling, we comprehensively mapped downstream targets and co‐regulatory networks. Our analysis defines BOLL‐dependent translational networks and demonstrates that BOLL forms functional, SDS‐resistant aggregates essential for coordinating meiotic mRNA translation. These results elucidate the mechanistic role of BOLL in human oogenesis and establish its aggregates as pivotal regulators of fertility, offering new avenues to diagnose and treat human reproductive disorders.

## 2 Results

### 2.1 Human BOLL Protein Is Specifically Expressed During Meiotic Prophase of Oogenesis

We first confirmed the expression pattern of BOLL in human female germ cells. Our results demonstrated that in humans, BOLL displays a distinctive complementary expression to DAZL in foetal germ cells, contrasting with the co‐expression observed in mice, where BOLL shows a transient peak at embryonic day 15.5 (E15.5), whereas DAZL is expressed continuously (Figure S1A,B). Quantitative colocalization analysis revealed stronger association between BOLL and meiotic progression in human germ cells, demonstrating significantly higher co‐expression frequency with the synaptonemal complex marker SYCP3 compared to DAZL (58.5% vs. 20.2%; Figure S1C). In contrast to humans, no mouse germ cells were found to co‐express Boll^+^ and Sycp3^+^ (Figure S1D), indicating that BOLL expression patterns diverge significantly between these species with human‐specific expression restricted to meiotic prophase I (Figure S1E,F). This divergence in expression patterns among paralogs aligns with prior reports describing their divergent roles during human oogenesis [1, 20].

To systematically explore the role of BOLL in cell cycle regulation, we established a differentiation system via BOLL overexpression under BMP4 treatment and enriched cells in the G2/M (4N) phase (Figure S2A). DNA content analysis at Day 6 revealed significantly elevated S‐phase (27.48% vs. vector control 12.08%) and 4N populations (44.02% vs. 27.26%) in BOLL‐overexpressing cells (Figure S2B). This 4N enrichment aligns with meiotic prophase I entry, consistent with prior human ovarian follicle induction study [21]. To detect whether cells derived from the in vitro differentiation system exhibited meiotic characteristics, we analysed the expression of meiosis‐specific genes in the 4N cell population on Day 6 by quantitative real‐time reverse transcription PCR (qRT‐PCR). Analysis of the 4N population revealed marked upregulation of meiosis‐related genes (*STRA8*, *ZGLP1*, *SYCP3*, *SYCP2* and *MEIOC*) and concomitant downregulation of pluripotency markers (*NANOG* and *OCT4*) in BOLL‐overexpressing cells (Figure S2C). Meiotic spreading and immunofluorescence staining for SYCP3 and γH2AX showed an elongated SYCP3 pattern in induced cell nuclei and reduced γH2AX in most nuclei with elongated SYCP3, indicating progression to a later meiotic stage (Figure S2D). These results demonstrate that BMP4 treatment combined with BOLL overexpression induces hESCs to differentiate into meiotic OLCs, effectively mimicking in vivo oogenesis stages, providing a functional platform for subsequent mechanistic dissection of BOLL‐mediated translational control during female gametogenesis.

### 2.2 BOLL Promotes Target mRNA Translation Through U‐Rich Motif Recognition in 3′UTRs

**FIGURE 1:**
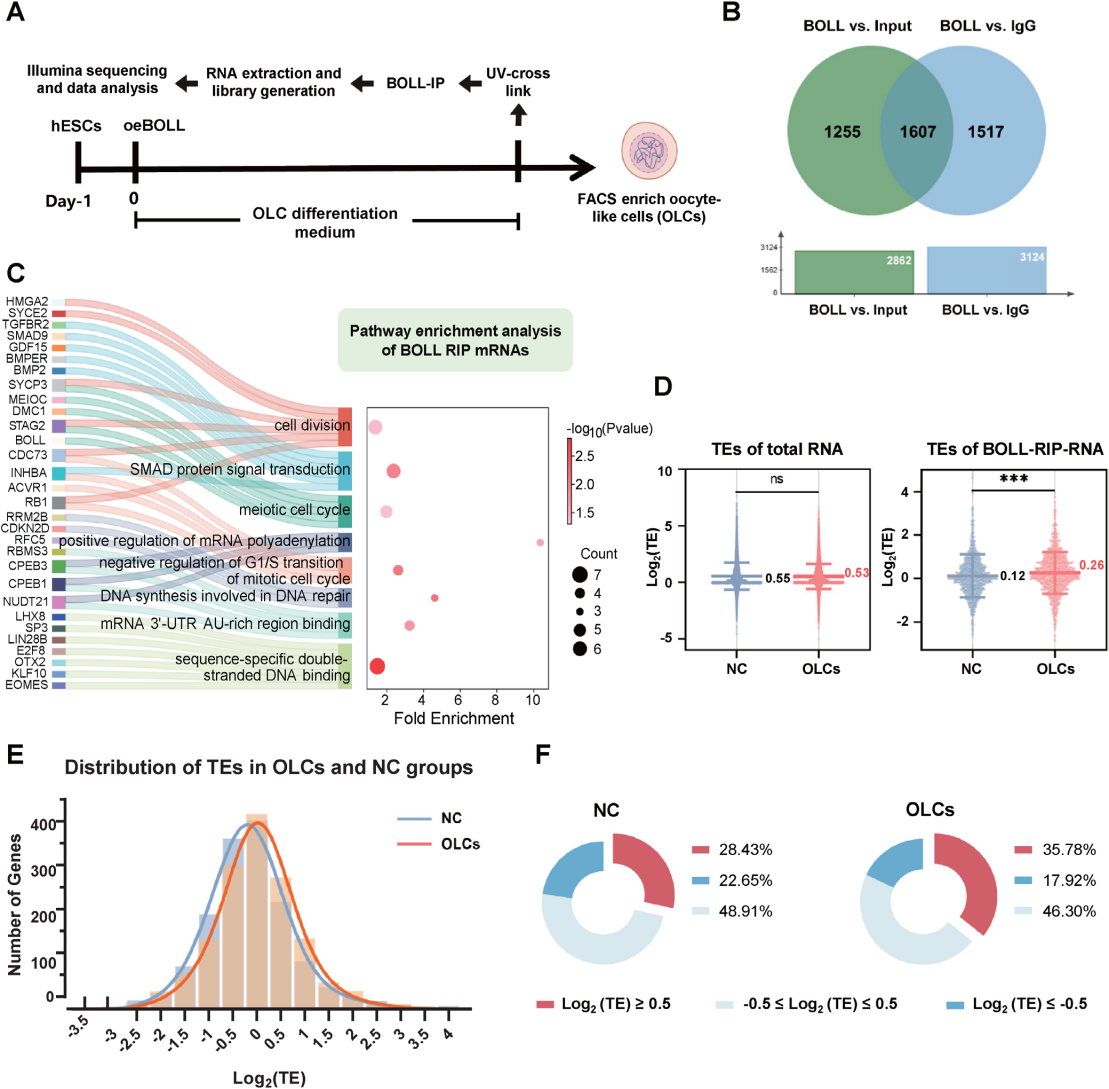
BOLL enhances the translation efficiency of target mRNAs in OLCs. (A) Schematic diagram of RIP‐seq experiment procedure. (B) Venn diagram depicting the genes significantly enriched in BOLL RIP compared to Input and IgG controls (Fold change (BOLL‐IP/IgG) > 2, Fold change (BOLL‐IP/Input) > 2, DEseq2, *p* < 0.05). (C) Sankey diagram and bubble chart illustrating the BOLL‐RIP‐RNA and the GO enrichment analysis. The Sankey diagram shows representative BOLL‐specific binding RNAs (total 1607), while the bubble chart displays the number of representative genes and enrichment fold changes in the enriched pathways (*p* < 0.05). (D) Comparison of the TE of the BOLL‐RIP‐RNA in OLCs and control group with empty vector overexpression. Significance was determined using Student's *t*‐test. ns, not significant; ***p < 0.001. (E) Frequency distribution histograms of the TE of BOLL‐RIP‐RNA in the OLCs group and the NC group with empty vector overexpression. Orange, OLCs group. Blue, NC group. (F) Pie charts of the TE of BOLL‐RIP‐RNA genes in the OLCs group and the NC group. Left panel represents the NC group; the right panel represents the OLCs group. The red portion indicates the proportion of RNA with high‐TE (Log2 (TE) ≥ 0.5), the blue portion indicates the proportion of RNA with low‐TE (Log2 (TE) ≤ −0.5), the light grey portion indicates the proportion of RNA with −0.5 ≤ Log2 (TE) ≤ 0.5.

To delineate BOLL‐associated molecular targets during meiotic prophase, we performed RNA immunoprecipitation sequencing (RIP‐seq) analysis in OLCs, identifying 1607 transcripts significantly enriched in the BOLL‐IP group compared to IgG controls (FDR < 0.05, |log2FC| > 2) (Figure 1A,B). Functional annotation revealed strong associations of BOLL‐bound RNAs with meiotic processes, including chromosome organisation, SMAD signalling, DNA repair synthesis and mRNA polyadenylation regulation (Figure 1C). Notably, key meiotic regulators (SYCE2, SYCP3, MEIOC, DMC1) were identified as BOLL targets, suggesting its role in post‐transcriptionally regulating meiosis‐associated genes. To investigate the translational regulatory role of BOLL in OLCs, we performed transcriptome‐translatome sequencing [22] and analysed the distinct translation efficiency (TE) patterns. BOLL‐enriched mRNAs exhibited significantly higher TE than controls (0.26 vs. 0.12), whereas global TE remained comparable between groups (0.53 vs. 0.55; Figure 1D). Consistent with this, BOLL‐RIP‐associated transcripts in OLCs showed right‐shifted TE distribution (Figure 1E), with increased high‐TE transcripts (35.78% vs. 28.43%) and decreased low‐TE transcripts (17.92% vs. 22.65%) relative to controls (Figure 1F). These results suggest that BOLL promotes meiotic protein synthesis through translational potentiation of mRNAs encoding cell cycle regulators.

**FIGURE 2:**
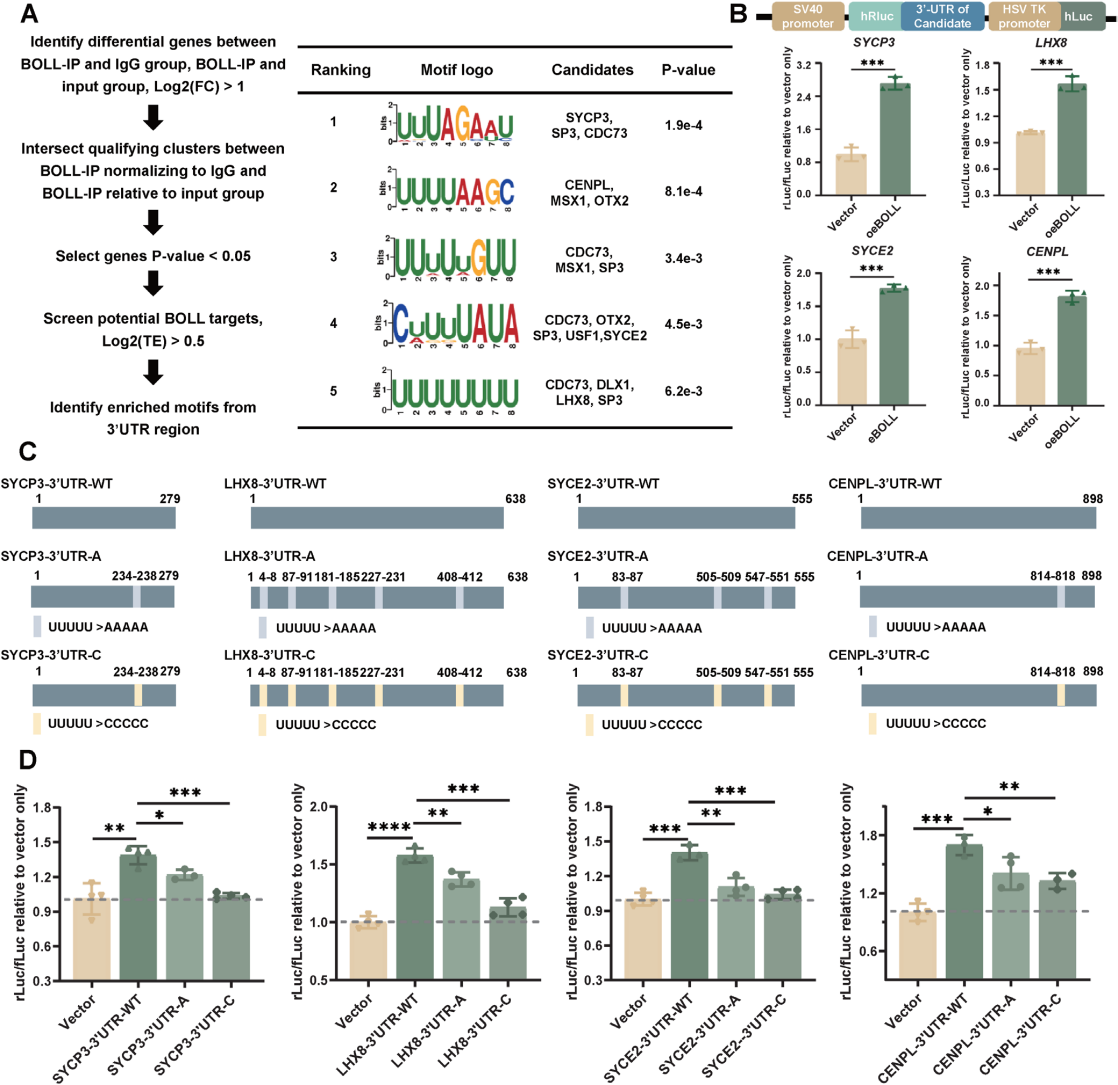
BOLL drives mRNA translation via U‐rich motif recognition in 3′UTRs. (A) Motif enrichment analysis from BOLL RIP‐seq. (B) 3′UTR luciferase reporter assays of BOLL target candidates in 293FT cells. Cells were transfected with empty vectors (NC) or BOLL‐overexpressing vectors. *n* = 3 (biological replicates from ~25,000 cells), three independent experiments conducted, ns, no significant difference, ***p < 0.001, calculated using Student's t‐test compared with Vector. (C) Schematic of 3′UTR U>A/U>C mutants for SYCP3, LHX8, SYCE2 and CENPL. Grey and yellow boxes mark U>A and U>C substitutions, respectively, at predicted BOLL‐binding sites. (D) Luciferase reporter assays for 3′UTR mutants in 293FT cells. Activity was measured after transfection with empty vector (NC), BOLL‐overexpressing vector or U>A/U>C mutants. Data include ≥ 3 biological replicates (each from ~25,000 cells) across three independent experiments. Student's *t*‐test: ns, not significant; *p < 0.01, **p < 0.001, ***p < 0.0001. Error bars indicate SD.

To depict the RNA binding site of BOLL, we utilised motif analysis using STREME on 3′UTR sequences from 615 high‐TE BOLL‐bound transcripts. This revealed conserved U‐rich elements (e.g., UUUAGAAU and UUUUAAGC; Figure 2A), aligning with known BOLL binding preferences [23]. Functional network analysis based on protein interactions revealed significant enrichment of BOLL targets in essential oogenic processes, including vesicle‐mediated transport, DNA replication initiation and meiotic cell cycle progression (Figure S3), corresponding to critical developmental events during human oogenesis. To validate the regulatory role of BOLL on candidate targets, we selected four mechanistically relevant targets: synaptonemal complex components SYCE2 and SYCP3, oogenesis regulator LHX8 [24, 25] and centromere assembly factor CENPL [26]. Dual‐luciferase assays demonstrated BOLL‐mediated translational enhancement through direct 3′UTR interaction, with SYCE2, SYCP3, LHX8 and CENPL showing significant activation compared to controls (Figure 2B). To further verify the recognised motif of BOLL, we generated mutant reporters by substituting conserved UUUUU sequences in 3′UTRs with AAAAA (3′UTR‐A) or CCCCC (3′UTR‐C) (Figure 2C). Data demonstrated a marked decrease in luciferase activity among the mutants, with the 3′UTR‐C group exhibiting a more substantial reduction than the 3′UTR‐A group (Figure 2D).

To define the specificity of BOLL‐RNA recognition, we used AlphaFold3 to predict the binding interface between BOLL and its target mRNAs. We examined two representative transcripts—SYCP3, whose 3′UTR contains a canonical U‐rich motif and LHX8, which harbours five tandem U‐rich motifs. Modelling of full‐length BOLL with the full‐length SYCP3 3′UTR positioned the RRM domain of BOLL adjacent to the U‐rich region of SYCP3 (Figure S4A), albeit with relatively low confidence. To improve accuracy and focus on the core recognition module, we modelled the BOLL RRM domain against a 21‐nt segment encompassing the U‐rich region of the LHX8 3′UTR. These predictions aligned with our functional assays, showing that the RRM domain contacted four of the five U‐rich motifs in LHX8 (Figure S4B). Mutating these motifs to AAAAA or CCCCC substantially reduced the predicted binding affinity. In particular, C‐rich mutations abolished three of the four interaction sites and weakened the remaining one (Figure S4C). Consistent with these observations, the computed binding free energy (Δ*G*) and dissociation constant (*K*_d_) supported a spontaneous and stable interaction between the BOLL RRM domain and U‐rich 3′UTR sequences (Figure S4D). Collectively, our experimental and predictive results support that BOLL drives translational activation of meiotic regulators through sequence‐specific recognition of U‐rich elements in 3′UTRs, providing a molecular framework for its essential role in prophase I progression during gametogenesis.

### 2.3 BOLL Interacts With Proteins Related to Translational Regulation

**FIGURE 3:**
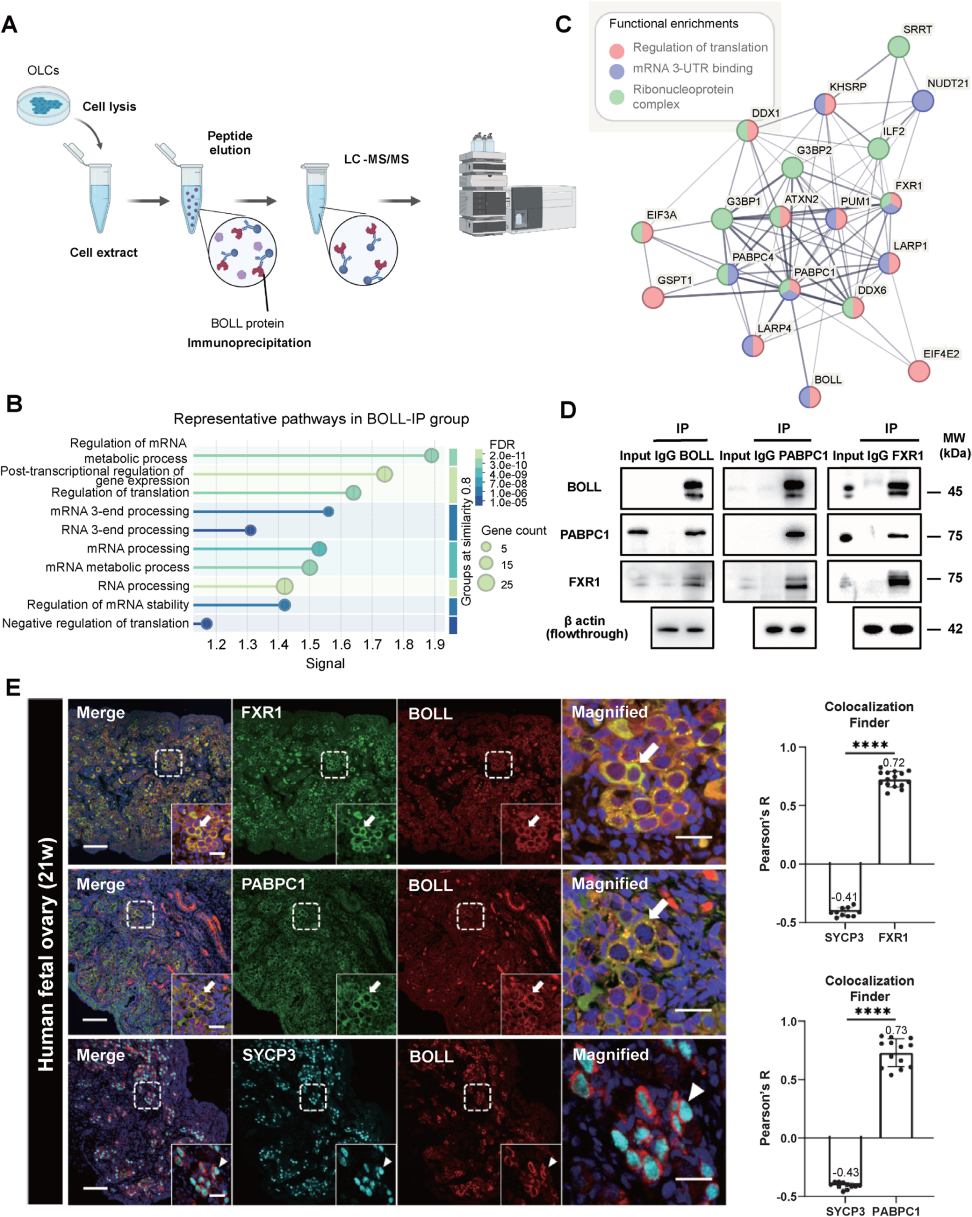
BOLL interacts with translation‐regulatory factors during human oogenesis. (A) Schematic illustration showing the workflow of proteomic analysis on BOLL‐IP and IgG‐IP proteins. (B) Representative GO enrichment analysis of BOLL‐IP proteins. Signal represents the weighted harmonic mean of the observed/expected ratio and −log_10_(FDR), where FDR (false discovery rate) indicates the statistical significance of enrichment. *p*‐Values were adjusted for multiple testing using the Benjamini–Hochberg procedure. (C) The network was analysed using the STRING database (version 12.0). Nodes represent proteins and lines represent interactions. Thick lines indicate experimentally validated direct physical interactions, while fine lines indicate indirect interactions based on prediction methods. (D) o‐IP experiment showing BOLL‐PABPC1‐FXR1 interaction in OLCs. β‐actin in the flow‐through following IP serves as an internal control. (E) Immunostaining of BOLL, FXR1, PABPC1 and DAZL in human foetal ovary (21 weeks), with nuclei counterstained with DAPI (blue). Middle panel shows representative enlarged images of female germ cells. White arrows indicate co‐localised signals, while triangles indicate non‐co‐localised signals. Right panel shows line plots of fluorescence intensity and Colocalization_Finder quantifying BOLL‐FXR1 or BOLL‐PABPC1 co‐localization. SYCP3 and BOLL co‐localization was used as a negative control. The Pearson correlation coefficient ranges from 0 to 1 for significant positive co‐localization and from −1 to 0 for no significant co‐localization. Scale bars: 100 μm in ×25 images, 20 μm in enlarged images.

To explore the protein–protein interaction network of BOLL, we conducted immunoprecipitation (IP) coupled with mass spectrometry (MS) on OLCs (Figure 3A) and identified 125 BOLL‐associated proteins (Figure S5A). GO enrichment analysis revealed that these proteins are primarily involved in mRNA metabolic regulation, translational control and mRNA stability regulation (Figure 3B), consistent with the conserved translational regulatory features observed in DAZ family proteins [27]. Protein interaction network analysis through STRING demonstrated that BOLL‐associated proteins mainly involved in cytoplasmic stress granule components, Clathrin‐coated pit and aminoacyl‐tRNA synthetase multi‐enzyme complexes (Figure S5B). Given that the role of BOLL in translation regulation, we further annotated proteins related to translation. Results showed BOLL‐binding proteins form three functionally interconnected modules: (1) translational regulators (e.g., EIF4E2, PABPC1), (2) mRNA 3′UTR‐binding (e.g., FXR1, PABPC4), (3) ribonucleoprotein complex (e.g., DDX6, G3BP2) (Figure 3C). Notably, PABPC1 (poly(A)‐binding protein) and FXR1 (Fragile X mental retardation‐related protein 1) participated in all three modules and both of them have been established as mediators of phase separation‐driven translational activation in male germ cells [28].

To validate these interactions, we performed co‐immunoprecipitation (co‐IP) assays in OLCs. The results confirmed the specific physical interactions between BOLL and both PABPC1 and FXR1 (Figure 3D). These associations remained stable following RNase A treatment (Figure S5C), indicating direct protein–protein interactions independent of RNA mediation. Further immunofluorescence analysis supported their physiological interaction in vivo meiotic germ cells, as evidenced by the distinct colocalization patterns between BOLL and both PABPC1 and FXR1 in 21‐week human foetal ovarian tissue (Figure 3E). These findings revealed that BOLL orchestrates the translational regulatory network during meiotic prophase I through specific binding to the regulatory elements of the translational complex.

### 2.4 BOLL Formed SDS‐Resistant Aggregates in the OLCs and Co‐Localised With Protein Aggregate Precursor Protein in Human Foetal Ovary

**FIGURE 4:**
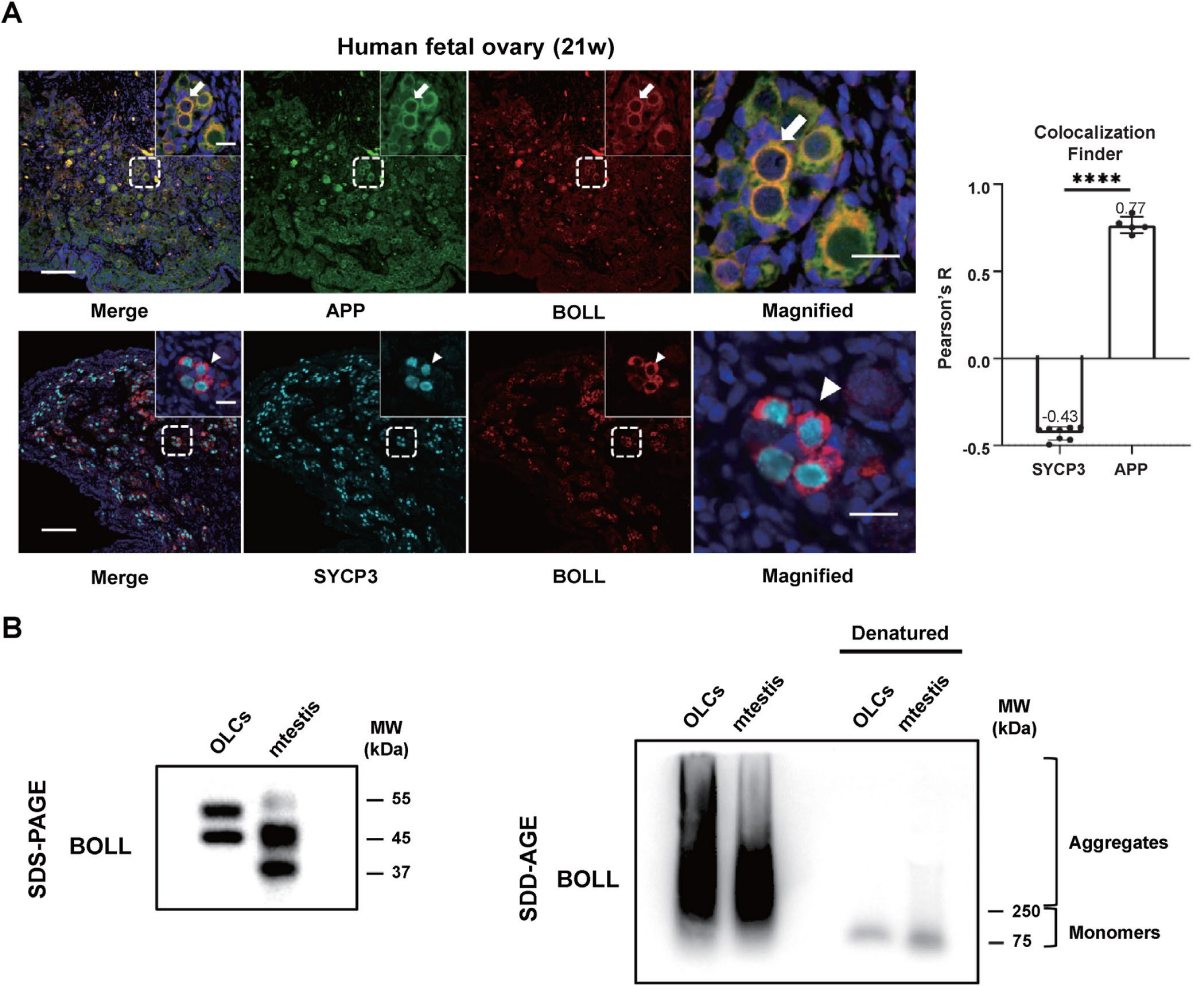
BOLL co‐localised with protein aggregate precursor in human foetal ovary and formed SDS‐resistant aggregates in the OLCs. (A) Immunofluorescence (IF) staining of human foetal ovary using anti‐BOLL (green) and anti‐APP (red) antibodies. BOLL and APP signals co‐localised in the cytoplasm. Middle panel shows enlarged images of female germ cells with white arrows indicating co‐localised signals. Right panel displays fluorescence intensity line plots and Colocalization_Finder analysis of BOLL‐APP co‐localization. SYCP3 and BOLL co‐localization served as a negative control. Scale bars: 100 μm (25× images), 20 μm (enlarged images). (B) SDD‐AGE and SDS‐PAGE analysis of OLCs and testes from P21 WT mice using anti‐BOLL antibody. Mouse testis extract was used as a positive control and denatured proteins indicated the monomer band.

Emerging evidence demonstrates the conserved role of RBPs in orchestrating gametogenesis through the assembly of functional protein aggregates, a mechanism critical for meiotic progression and post‐transcriptional regulation [10, 29]. A study in mouse testes has demonstrated that mouse Boll forms functional aggregates in male germ cells [18]. To investigate whether human BOLL exhibits similar aggregation properties, we analysed its colocalization with amyloid precursor protein (APP), a marker of aggregated protein structures, in 21‐week foetal ovarian tissue. The results demonstrated a clear co‐localization relationship between BOLL and APP in germ cells, with a Pearson correlation coefficient of 0.77 (Figure 4A), indicative of spatial and functional association.

To further characterise BOLL aggregation, we employed semi‐denaturing detergent agarose gel electrophoresis (SDD‐AGE), a method widely used to detect SDS‐resistant aggregates in neurodegenerative disease models and yeast meiosis [9, 29, 30, 31]. SDD‐AGE analysis of OLCs revealed high‐molecular‐weight BOLL aggregates (> 250 kDa), distinct from the predicted monomeric forms (37–55 kDa) observed in SDS‐denatured mouse testicular lysates (Figure 4B). These findings demonstrate the capacity of human BOLL to form SDS‐resistant aggregates in vitro, similar to the biochemical behaviour of its murine ortholog.

Given that protein aggregation can be driven by liquid–liquid phase separation (LLPS) [10], we investigated whether BOLL exhibits LLPS activity. In sections of 21‐week human foetal ovary, BOLL was observed to form distinct punctate structures within a subset of germ cells (Figure S6A), indicating that BOLL protein undergoes compartmentalization into puncta‐like structure in vivo. Moreover, SDD‐AGE assays performed in 293FT cells confirmed the presence of BOLL polymeric assemblies (Figure S6B). To visualise their aggregation in the cells, we constructed BOLL and monomer green fluorescent protein (mGFP) fusion constructs and transfected them into 293FT cells. And the fluorescence recovery after photobleaching (FRAP) analysis was used to determine the dynamics and mobility of phase‐separated liquid droplet or condensate in live cells. Notably, BOLL formed punctate condensates and these condensates recovered over 70% of the fluorescence signal within approximately 170 s after photobleaching (Figure S6C). These results demonstrate that BOLL undergoes LLPS and forms dynamic condensates within cells. Interestingly, these protein aggregates resemble the unique polymeric structures observed during yeast meiosis [32], suggesting a conserved mechanism through which eukaryotic cells modulate the biophysical properties of subcellular compartments via phase separation to ensure accurate segregation of genetic material during gametogenesis.

### 2.5 DAZ‐Containing Domain Is Essential for the Formation of BOLL Aggregates

**FIGURE 5:**
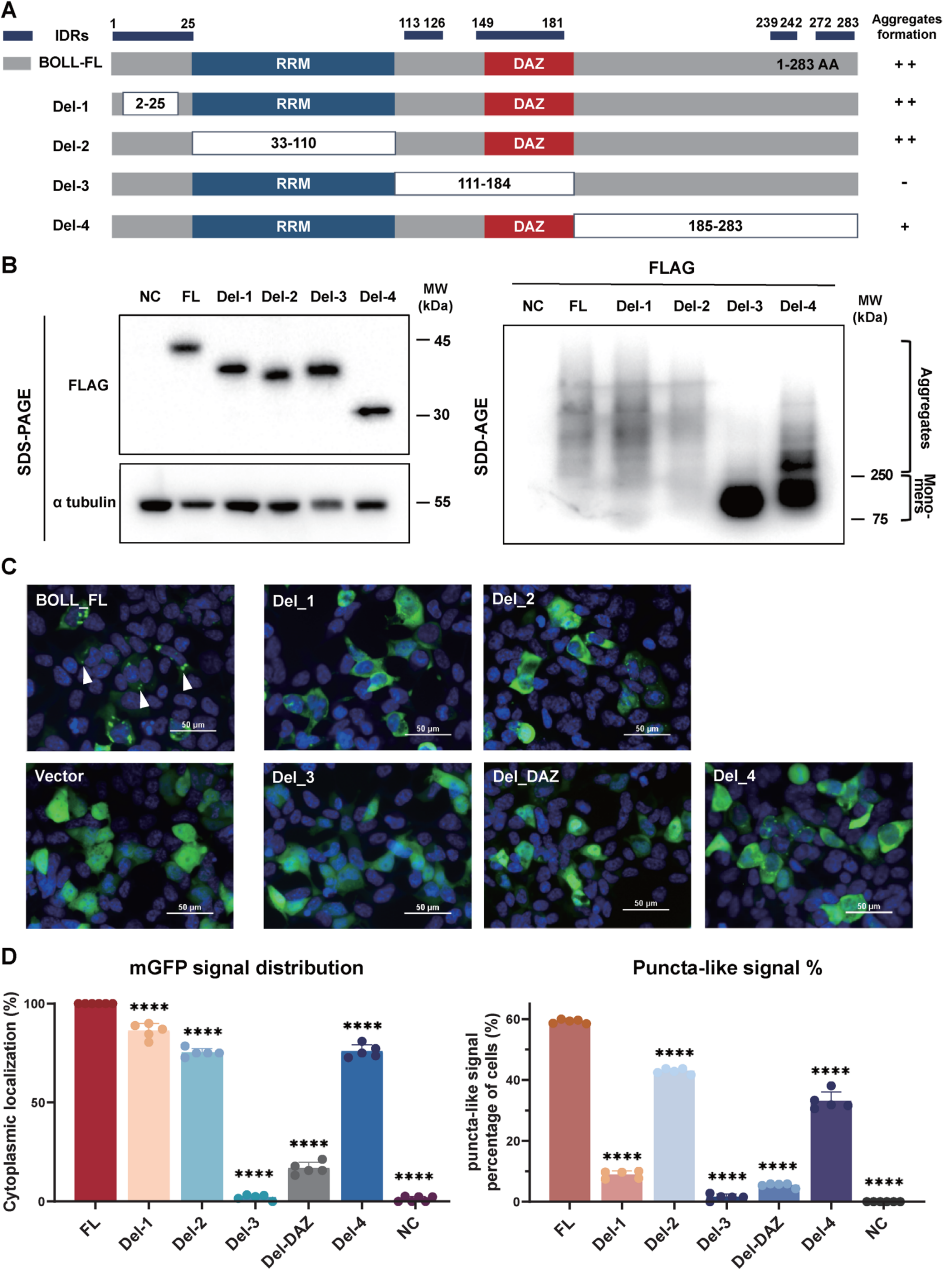
DAZ‐containing domain of BOLL is essential for protein aggregation. (A) Diagram of BOLL deletion constructs. The blue line denotes the intrinsically disordered regions (IDRs) identified via MobiDB (https://mobidb.bio.unipd.it/). (B) SDD‐AGE and SDS‐PAGE analysis of full‐length BOLL (p2k7‐BOLL‐FL) and its variants using anti‐FLAG antibody. p2k7‐BOLL deletion plasmids (Del‐1 to Del‐4) and p2k7‐BOLL‐FL plasmid were packaged into lentiviruses and used to infect differentiated hESCs. p2k7‐vector alone served as a negative control. After 6 days, cells were collected for assays to detect BOLL aggregates and monomers via anti‐FLAG and anti‐α‐tubulin antibodies. RRM, RNA Recognition Motif and DAZ, Deleted in Azoospermia domain, a protein–protein interaction domain. (C) Fluorescence imaging of full‐length and truncated BOLL proteins. 293FT cells were transfected with either an empty pCDNA3.1‐mGFP vector (control), pCDNA3.1‐BOLL‐FL‐mGFP (full‐length) or pCDNA3.1‐BOLL‐Del‐mGFP (truncations Del‐1 to Del‐4). Representative images were captured 48 h post‐transfection to assess changes in punctate structure formation and subcellular localization. White triangles mark the puncta‐like signal sites. Scale bar, 50 μm. (D) Quantitative analysis of BOLL puncta‐like signals and subcellular localization. Statistical analysis of cells containing BOLL puncta‐like signals (left), showing the proportion of transfected cells containing distinct mGFP‐tagged protein with puncta‐like signals. Data are from three independent experiments. Quantification of subcellular localization for full‐length and truncated BOLL (right), indicating the percentage of cells exhibiting cytoplasmic localization was calculated. Cells displaying any nuclear signal were excluded from the cytoplasmic count. Statistical significance was determined by Student's *t*‐test (****p ≤ 0.0001).

Previous studies have reported that low complexity domains (ICDs) or intrinsically disordered regions (IDRs) drive LLPS through weak, multivalent interactions, a process integral to diverse cellular functions [8, 33, 34]. LLPS has been implicated in initiating protein aggregation, potentially underlying early stages of BOLL aggregate formation in mice [18]. Based on these findings, we investigated the role of IDRs in human BOLL protein aggregation. Using the MobiDB algorithm, we mapped IDR distribution across the BOLL sequence, identifying key disordered segments (Figure 5A).

To delineate regions critical for aggregation, we generated four FLAG‐tagged BOLL truncation constructs (Del‐1 to Del‐4). SDD‐AGE analysis revealed that deletion of residues 111–184 (Del‐3) abolished aggregate formation, pinpointing this region as essential for SDS‐resistant aggregation (Figure 5B). The truncation of the C‐terminal 99 residues downstream of the DAZ domain (Del‐4) partially impaired aggregation, with co‐existing aggregated and monomeric forms. In contrast, Del‐1 and Del‐2 truncations exhibited no significant effect on aggregation, underscoring the specificity of the Del‐3 region.

To investigate the contribution of distinct domains to BOLL aggregation and localization, full‐length and various truncated (Del‐1 to Del‐4, Del‐d) BOLL‐mGFP fusion plasmids were constructed and expressed in 293FT cells. It was observed that full‐length BOLL‐mGFP formed distinct cytoplasmic puncta in approximately 60% of cells (Figure 5C,D). In contrast, the nuclear localization frequency increased to 10%–25% for Del‐1, Del‐2 and Del‐4, while the proportion of cells forming puncta decreased by 20%–30% for Del‐2 and Del‐4. For Del‐1, which lacks the N‐terminal IDR, the puncta‐formation rate was further reduced to about 8% (Figure 5C,D). Notably, Del‐3, lacking the DAZ domain and its upstream disordered region, completely lost the ability to form puncta and exhibited diffuse nuclear localization (Figure 5C). Similarly, Del‐d, which lacks only the DAZ domain, was also predominantly localised to the nucleus, with only about 17% of cells displaying cytoplasmic fluorescence and the proportion of cells with puncta was markedly reduced to approximately 5% (Figure 5D). These results suggest that the region containing the DAZ domain plays a critical role in the proper subcellular localization and phase‐separation‐driven condensation ability of BOLL.

To further identify the critical peptide region for BOLL aggregate formation, we subdivided the Del‐3 into four shorter truncations (Del‐a to Del‐d; Figure S7A). The expression levels of these truncated proteins were normalised using SDS‐PAGE. SDD‐AGE analysis confirmed that residues 111–184 are indispensable for aggregation. While the Del‐a to Del‐d truncations mildly impaired aggregate formation, high‐molecular‐weight BOLL aggregates were still detectable (Figure S7B). Furthermore, additional truncation analysis of the Del‐4 region revealed that the C‐terminal IDR‐rich segment (residues 235–283) also modulates aggregation efficiency (Figures S7C and S4D), suggesting a cooperative interplay between structured and disordered regions during BOLL assembly. Together, these findings collectively identify residues 111–184 as the primary determinant of BOLL aggregation, supporting a conserved mechanism through which aggregation‐prone regions direct the assembly of protein complexes during gametogenesis.

### 2.6 BOLL Aggregates Are Indispensable for the Recruitment of Interacting Proteins and Translational Regulation of Target mRNAs

**FIGURE 6:**
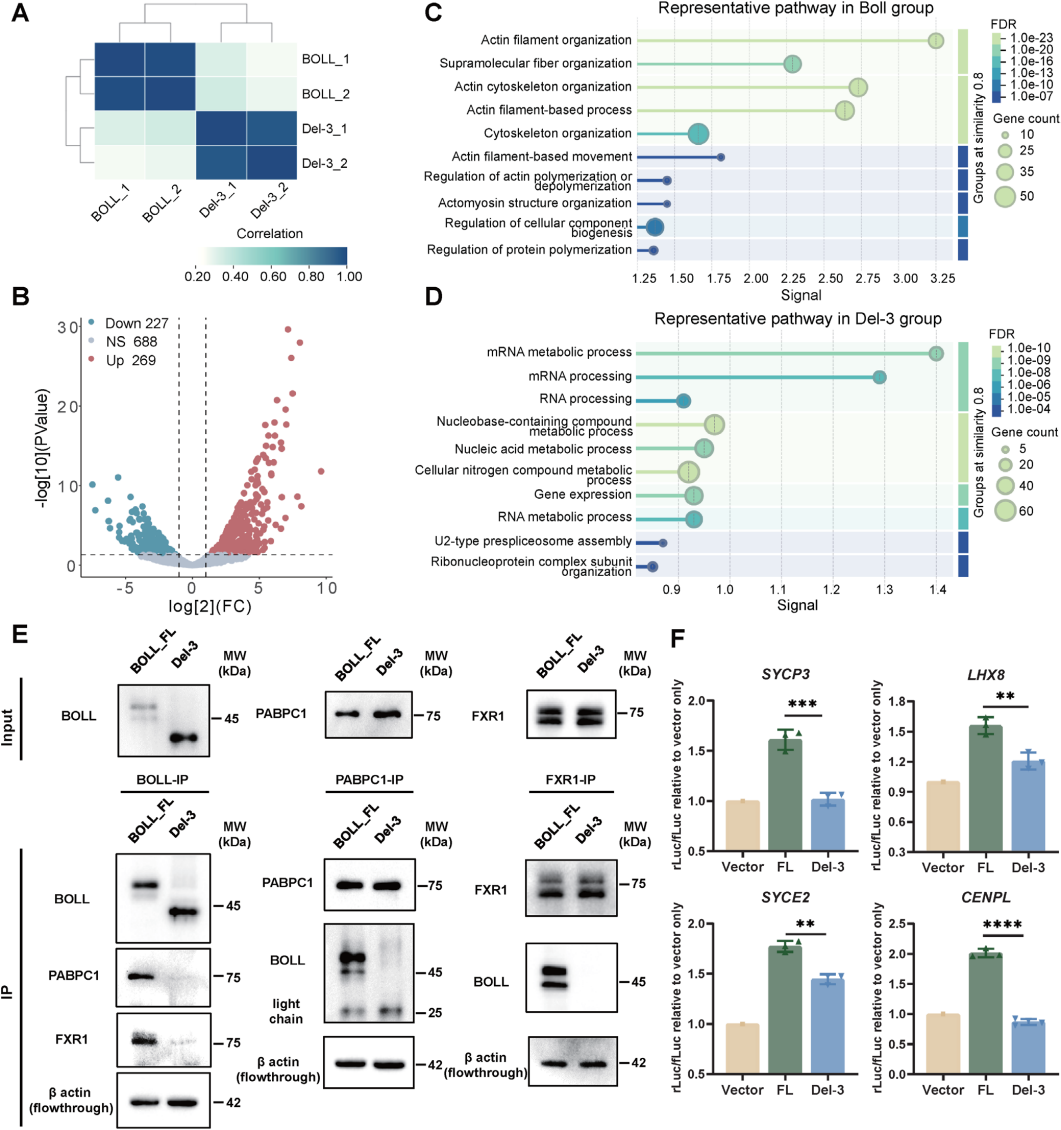
Disruption of BOLL aggregates impairs its interactome assembly and translational regulation of target genes. (A) The spearman correlation coefficient among four IP‐MS analyses for proteins enrichment in BOLL group and Del‐3 group with two replicates. (B) Volcano plot identifying differentially enriched proteins (*p* < 0.05; |fold‐change| > 2) between BOLL and Del‐3 immunoprecipitations. Anti‐BOLL‐enriched (red) and anti‐Del‐3‐enriched (green) proteins are highlighted. Dashed lines indicate significance thresholds. (C, D) Representative GO terms enrichment of BOLL‐specific interactors (C) and Del‐3‐associated proteins (FDR < 0.05 by Fisher's exact test with Benjamini‐Hochberg correction). (E) co‐IP experiment validation showing BOLL interacts with PABPC1 and FXR1. Del‐3 exhibits significantly impaired interaction with these proteins. β‐actin serves as loading control. (F) Dual‐luciferase reporter assays demonstrating Del‐3 impaired the regulatory capacity on 3′UTRs of SYCP3, SYCE2, LHX8 and CENPL. ns, no significant difference, **p < 0.01, ***p < 0.001, ****p < 0.0001. Significance analysis between the FL and Del‐3 groups was performed using Student's *t*‐test.

To investigate the protein composition of BOLL aggregates, we performed comparative proteomic analysis between full‐length BOLL and its truncated variant (Del‐3). Distinct proteomic profiles were observed between the two groups (Figure 6A). Specifically, 269 proteins showed significant enrichment in the full‐length BOLL group, while 227 proteins were uniquely associated with the Del‐3 truncation (Figure 6B). Functional annotation of BOLL full‐length–enriched proteins revealed strong associations with actin cytoskeleton dynamics, including actin filament organisation, supramolecular fibre assembly and regulation of protein polymerisation (Figure 6C). These findings suggest that BOLL aggregates exhibit multimeric properties dependent on structural proteins. In contrast, Del‐3 associated proteins were predominantly linked to nuclear RNA processing pathways, such as mRNA metabolic regulation, gene expression and U2‐type prespliceosome assembly (Figure 6D), implying aberrant nuclear interactions that may competitively inhibit cytoplasmic RNA processing machinery.

Protein interaction network analysis demonstrated that full‐length BOLL‐associated proteins cluster into functional hubs involving mRNA processing, stress granule assembly and actin cytoskeleton organisation (Figure S8A). Notably, actin filament branching proteins (ARP2/3 complex) and actin regulators (Rho GTPase family) were prominently enriched, implying actin structural contribution to BOLL aggregates. In contrast, Del‐3‐interacting proteins formed networks involved in spliceosome function, DNA replication and RNA Pol II elongation complexes (Figure S8B). These results demonstrated that the truncation in Del‐3 impairs its ability to recruit polymerisation‐regulating partners, disrupting its ability to form functional aggregates.

To determine whether BOLL aggregate formation relies on cytoskeletal architecture, we treated OLCs with Cytochalasin D (microfilament inhibitor) or Nocodazole (microtubule inhibitor). While immunofluorescence confirmed effective disruption of microfilament and microtubule networks (Figure S9A), SDD‐AGE and SDS‐PAGE analyses revealed no significant alteration in BOLL aggregate formation (Figure S9B). These results indicate that BOLL aggregate assembly is independent of cytoskeletal scaffolds, consistent with prior in vitro findings demonstrating that murine Boll protein spontaneously forms SDS‐resistant aggregates at physiological concentrations [18].

To assess the impact of BOLL truncation on co‐factor recruitment, we performed co‐IP assays in OLCs expressing full‐length BOLL or Del‐3. Full‐length BOLL demonstrated robust interactions with translational regulators FXR1 and PABPC1, whereas Del‐3 exhibited markedly diminished binding capacity (Figure 6E). These results underscore that the structural integrity of BOLL aggregates is essential for recruiting translational machinery. Together, our data demonstrate that the AA111–184 region is required both for BOLL aggregate assembly and for direct interaction with key translational regulators, including FXR1 and PABPC1.

To further verify the functional impact of this interaction deficit, we employed a dual‐luciferase reporter system fused to the 3′UTRs of key germ cell genes (SYCE2, SYCP3, CENPL and LHX8). Overexpression of Del‐3 significantly reduced luciferase activity compared to full‐length BOLL across all target 3′UTRs (Figure 6F). Notably, Del‐3 abolished the regulatory effects on SYCP3 and CENPL, reducing luciferase activity to baseline. Our results demonstrate that the Del‐3 protein, which disrupts protein condensates, exhibits a compromised ability to interact with translation‐related proteins, thereby affecting its capacity to promote target gene translation.

To elucidate the structural basis of BOLL interaction with translational regulators, we employed AlphaFold3 modelling to predict binding interfaces between BOLL and FXR1‐PABPC1 complexes. The model revealed specific hydrogen‐bonding interactions between the DAZ domain of BOLL and the KH1‐KH2 tandem domains of FXR1. Flexible interactions were observed between IDR residues (T149/S120) of BOLL and conserved motifs (Q60 in RRM domain, E587 in PABC domain) of PABPC1, while the RRM domain of BOLL showed minimal participation in protein–protein interactions (Figure S10A). This observation aligns with experimental evidence demonstrating intact aggregate formation in RRM deletion mutants, suggesting RNA‐independent assembly mechanisms similar to FXR1‐mediated translational activation through phase separation [28]. Structural modelling of the Del‐3 truncation variant (Δ111–184) revealed peripheral localization within the predicted complex (Figure S10B), with disrupted binding interfaces for both FXR1 and PABPC1. These computational predictions corroborate experimental findings showing impaired aggregate assembly and protein recruitment capacity in Del‐3 mutants, underscoring the essential role of these structured domains in biomolecular condensate formation.

## 3 Discussion

During the meiotic phase of oogenesis, germ cell‐specific RBPs play a crucial role in the translational regulation of transcripts [1, 35, 36]. Previous studies have confirmed the importance of BOLL in meiotic progression in lower organisms such as *Drosophila melanogaster* and *C. elegans* [37, 38, 39]. Our research reveals its specific role in translational control during human oogenesis. Mechanistic investigations demonstrate that BOLL forms functional protein aggregates capable of recruiting translational machinery components such as PABPC1 and FXR1, thereby activating target mRNAs critical for germ cell development. We propose a model that BOLL expression increases progressively in human oocytes during meiotic prophase I, driving aggregate formation that facilitates interaction with translation regulators to promote the synthesis of oogenesis‐related proteins.

The evolutionary trajectory of BOLL presents intriguing parallels and divergences. Although BOLL mutations remain rare in human infertility cases, reduced transcript levels correlate with male reproductive dysfunction [40]. The scarcity of reported mutations in females may reflect sampling limitations combined with functional compensation by its homologue DAZL, which exhibits multiple infertility‐associated variants [41, 42, 43]. Notably, Drosophila lacks DAZL orthologs but harbours Boll mutants (GenBank accessions NM_079265, NM_168318‐20) [40], suggesting evolutionary divergence where DAZL assumes BOLL functions in higher organisms. Further supporting this divergence, ablation of boll in dazl‐deficient species ( *S. mediterranea* and *C. elegans* ) induces germ cell meiotic arrest and oocyte depletion [16, 17], indicating the essential role of boll meiotic progression. In contrast, our experimental system relies on BOLL overexpression to generate OLCs due to the absolute requirement of exogenous BOLL for meiotic initiation. This technical constraint currently precludes loss‐of‐function validation in human germ cells. The transient expression pattern of BOLL in females and its co‐expression with DAZL in males may provide dual protective mechanisms against genetic perturbations, ensuring fidelity in germline development.

The biophysical basis of BOLL condensate formation warrants further exploration. Our data exclude cytoskeletal components like actin or microtubules as direct mediators of aggregation. Instead, the IDRs within BOLL likely enable multivalent interactions that drive concentration‐dependent aggregation, which is supported from in vitro observations that purified mouse BOLL undergoes spontaneous aggregation at specific concentrations [18]. In summary, aggregate formation is a complex process involving specific protein domains, cellular environments and protein–protein interactions. Future research should utilise immunofluorescence in conjunction with time‐lapse imaging methods to elucidate the spatiotemporal regulation of the aggregation throughout meiotic progression.

The formation of biomolecular aggregates with translation‐regulating functions appears to be an evolutionarily conserved strategy in gametogenesis. While studies in budding yeast have identified RBP assemblies essential for meiotic translation control [10] and similar structures have been observed in mammalian systems [18], our discovery of BOLL‐containing aggregates in human oogenesis extends this paradigm to primates. These findings parallel the LLPS‐mediated mechanism of FXR1 in spermiogenesis, where phase‐separated granules coordinate mRNA activation during late spermatid development [28]. The functional conservation underscores the critical role of dynamic RBP assemblies in germ cell development across species.

Based on the functional association between BOLL aggregates and cell cycle‐related gene regulation, we propose several potential mechanisms underlying their biological roles: (1) ribosomal recruitment capacity, as evidenced by MS identification of ribosomal subunit components. The aggregate structure may directly engage translation machinery through BOLL‐mediated interactions with ribosomes and target mRNAs, enabling coordinated synthesis of meiotic cell cycle regulators and oocyte developmental proteins. (2) BOLL aggregates may serve as a reservoir for protein storage. Given the dynamic autophagy occurring throughout meiosis [44], BOLL aggregates may function as temporary storage compartments for critical meiotic progression factors including CDK1, actin‐associated proteins and Rho GTPases through filamentous bundling. This storage mechanism aligns with evolutionary conserved strategies observed in human oocyte filamentous lattices [45]. (3) BOLL aggregates may constitute a cytoprotective mechanism. Proteomic analysis reveals significant enrichment of stress granule‐associated proteins (e.g., DDX6, ATXN2 and FMR1) within BOLL aggregates. The aggregate architecture may enhance mechanical resilience to withstand environmental stressors during meiotic chromosomal remodelling [46, 47]. While our findings establish functional significance for BOLL aggregates in oogenesis, certain limitations warrant future investigation. The in vitro experimental system may not fully recapitulate the complexity of in vivo gametogenesis. Subsequent studies employing correlative light‐electron microscopy (CLEM) of cryopreserved clinical specimens could visualise native BOLL aggregation patterns in germ cells while mapping interactions with supramolecular fibres and ribosomal complexes. Advanced imaging modalities coupled with biophysical analyses should elucidate the structural dynamics and material properties of these assemblies.

This study establishes BOLL‐mediated translational regulation as an evolutionarily conserved mechanism in gametogenesis, bridging insights from model organisms to human reproductive biology. Functional parallels between BOLL aggregates and FXR1 phase‐separated granules underscore the fundamental importance of biomolecular condensation in germ cell development. However, the transient expression profile of BOLL in female gametes and technical limitations in human oocyte research necessitate innovative approaches. Generating aggregation‐deficient BOLL variants without altering protein expression levels will help disentangle condensate‐specific functions from general protein activity. Ultimately, deciphering the biophysical principles governing these dynamic assemblies may reveal novel therapeutic targets for infertility management.

## 4 Conclusion

In conclusion, our study defines a mechanism whereby the meiotic protein BOLL acts as a translational hub to direct mRNA activation in the human germline. We establish that BOLL binds U‐rich elements within 3′UTRs and recruits the effector proteins FXR1 and PABPC1, a complex we validated in human foetal germ cells that drives the translation of target mRNAs.

We further demonstrate that BOLL forms SDS‐resistant aggregates dependent on its DAZ domain and N‐terminal disordered regions. This aggregation is essential for its function, as it enables proper subcellular localization and the efficient recruitment of translational machinery.

**FIGURE 7:**
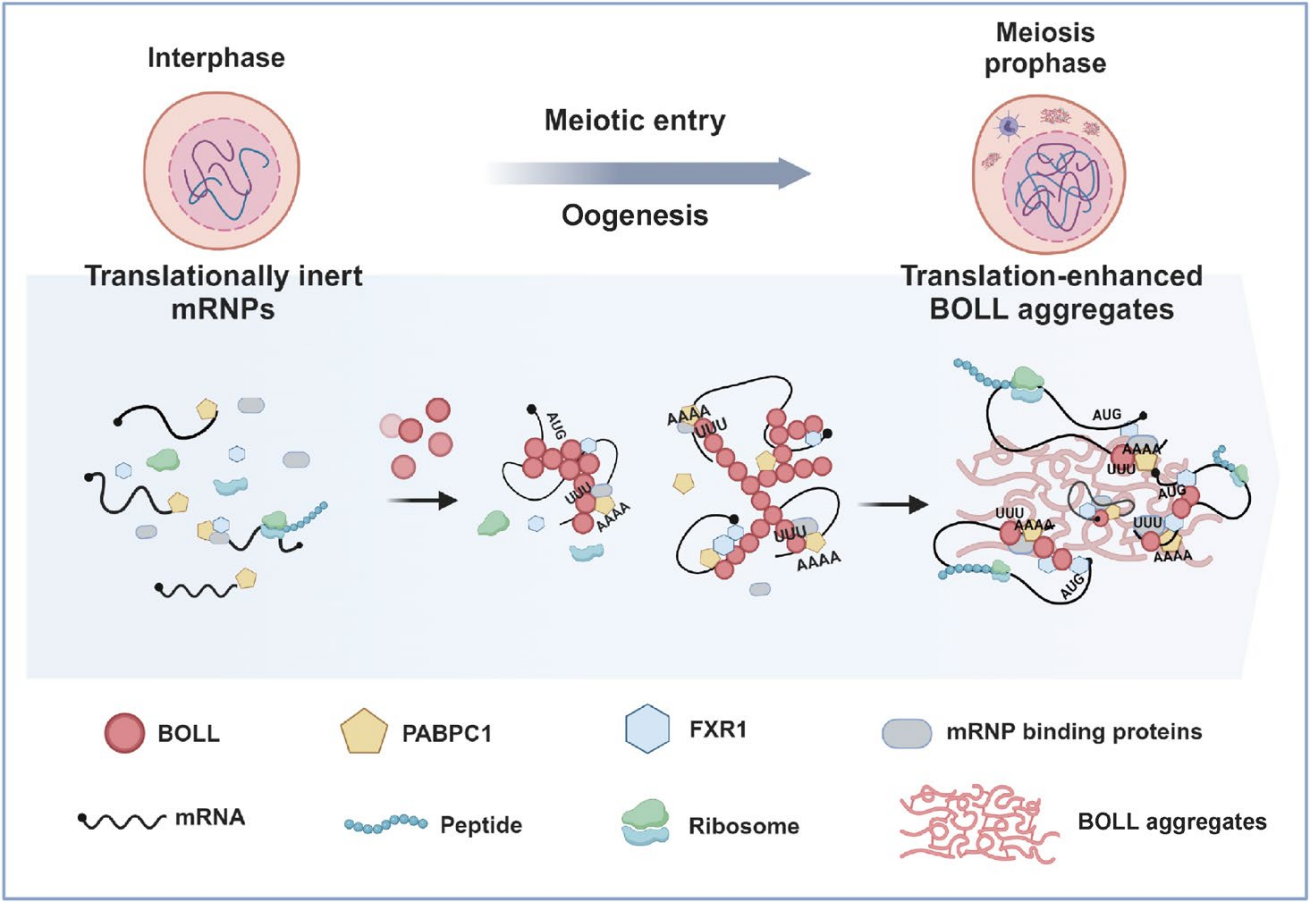
BOLL‐containing aggregate mediates translational regulation during human oogenesis. Schematic model showing that BOLL promotes the translation of oogenesis‐related mRNAs in oocyte‐like cells through protein aggregates by interacting with components of translation machinery (created with BioRender.com).

Based on these findings, we propose a model wherein the meiotic‐specific expression and aggregation of BOLL establish a translational condensate that concentrates meiosis‐related mRNAs and effector proteins. This system ensures the robust synthesis of key factors such as SYCE2 and SYCP3, thereby facilitating oogenesis (Figure 7). Our study underscores a conserved post‐transcriptional regulatory mechanism orchestrated by germ cell‐specific RBPs.

## 5 Materials and Methods

### 5.1 Cell Culture

The hESC line H9 (female, XX) was purchased from WiCell Inc. and cultured on a feeder layer of mouse embryonic fibroblasts in Knockout DMEM (Thermo Fisher, USA) with 20% KnockOut Serum Replacement (Thermo Fisher, USA), 25 μg/mL bFGF (Gibco, USA), penicillin–streptomycin (Gibco, USA), GlutaMAX (Gibco, USA), NEAA (Gibco, USA). The 293FT cell was cultured in high glucose DMEM (Thermo Fisher, USA) with 10% foetal bovine serum (FBS; Gemini, USA), penicillin–streptomycin, GlutaMAX, sodium pyruvate, NEAA.

### 5.2 Immunofluorescence Staining

Immunofluorescence staining was conducted following a previously described protocol [48]. In brief, cells were incubated in a suitable petri dish. Then the differentiated cells were sequentially washed three times with PBS. After being fixed with 4% paraformaldehyde for 10 min, the cells were rinsed with PBS three times. Then the cells were permeabilized with 0.25% Triton X‐100 for 10 min. Subsequently, the samples were incubated with specific primary antibodies. The primary antibodies used included Mouse anti‐DAZL (Bio‐Rad, MCA2336), BOLL Polyclonal antibody (Proteintech, 13720‐1‐AP), Rabbit Polyclonal SYCP3 Antibody (Novus, NB300‐232), FXR1 Polyclonal Antibody (Proteintech, 13194‐1‐AP), PABP Polyclonal Antibody (Abcam, ab21060). Each primary antibody was diluted at 1:1000. Then the cells were stained with secondary antibody using donkey anti‐mouse 488 (Invitrogen, A21202) or Alexa Fluor 594 (Invitrogen, A21203) for 1 h in the dark. After mounting with DAPI‐containing mounting medium (Sigma‐Aldrich, D9542) for 10 min, the cells were imaged using a confocal laser scanning microscope (FV3000), which enabled visualisation and analysis of the localization and distribution of specific proteins within cells.

Meiotic spreads were conducted as described previously with some modifications [49, 50]. Induced hESCs were collected as a single cell suspension from one well of a six‐well plate, centrifuged, resuspended in hypotonic extraction buffer and dropped onto slide glasses dipped in 1% paraformaldehyde. The slide glasses were kept overnight in a humidified box at room temperature. The slides were washed in water containing 0.04% Photoflow (Kodak) and completely dried at room temperature. The dried spreads were incubated with blocking buffer for 30 min at room temperature. The spreads were then incubated with the indicated primary antibodies listed in Table S1 at 4°C overnight followed by washes with TBS and an incubation with the indicated secondary antibodies for 1 h at room temperature. The slides were observed under a confocal microscope (Nikon).

### 5.3 Plasmid Construction and Overexpression

#### 5.3.1 Overexpression Vector Construction and Lentivirus Production

Overexpression vectors of BOLL were constructed and tested previously [51]. Overexpression vectors carrying EF1α promoter followed by cDNA of candidate genes were constructed using the Gateway system (Invitrogen, USA) as previously described [52, 53]. Lentivirus vector p2k7 without cDNA following EF1α promoter was set as a control for transduction. Lentivirus of six candidate factors or p2k7 was produced with the 293FT cell line transfected with vectors of VsVg, △8.9 and overexpressing vectors by Lipofectamine 2000 (Invitrogen).

### 5.4 Plasmid Construction and Transfection

For BOLL expression using pCDNA3.1, segments of the human BOLL were subcloned into the pCDNA3.1 vector by using HindIII and XbaI restriction enzymes (New England BioLabs Inc.). Plasmid pCDNA3.1‐hBOLL‐FL‐mGFP and human BOLL deletion plasmids (Del‐1 to Del‐4, Del‐d) were generated by PCR amplification of the CDS followed by recombining into pCDNA3.1‐Entry destination plasmid (Invitrogen, USA) using the ClonExpress MultiS One Step Cloning Kit. Hela cells in a 3.5 cm glass dish plate were transfected with 1 μg of pCDNA3.1 construct carrying either segment of BOLL using ExFect Transfection Reagent. Droplets were observed 24 h later on a glass‐bottom cell culture dish for fluorescence imaging.

### 5.5 Quantitative Real‐Time Reverse Transcription PCR

Total RNA was extracted with TRIZOL. Reverse transcription was performed with cDNA synthesis supermix (TRANSGEN, China). Quantitative real‐time PCR (qPCR) was performed and analysed under the manual instruction of TransStart Green qPCR SuperMix kit (TRANSGEN), Bio‐Rad CFX Connect instrument (Bio‐Rad, USA) and Biorad‐CFX manager software. Relative expression of genes normalised with *GAPDH* was calculated with the ΔΔ*C*q method. Primer sequences are listed in Table S2.

### 5.6 DNA Content Analysis

hESCs were first seeded at 50% confluency onto six‐well plates coated with Matrigel. To induce differentiation, adherent cells were treated with 50 ng/mL BMP4 for 1 h in differentiation medium and followed by transduction of lentivirus expressing BOLL for 1 day, recovery for 1 day, selection with blasticidin at 2 μg/mL for 3 days and recovery for another day. Thereafter, cells were treated with differentiation medium until Day 8. Differentiated cells were collected into a 15 mL tube, washed once with 1 mL chilled PBS and suspended in 5 μg/mL Hoechst 33342 (Beyotime) and 20 μM verapamil hydrochloride (Sigma). Cell suspension was incubated at 37°C for 40 min. During the incubation, tubes were mixed several times. The cells were resuspended in 20 μM verapamil hydrochloride (Sigma) and analysed by LSRFortessa SORP (BD). A total of > 500,000 cells from each sample were subjected to DNA content analysis using programmes in FlowJo 7.6 software package.

### 5.7 RIP‐Seq

1,000,000 FACS harvested OLCs were in lysis buffer (50 mM Tris–HCl, pH 7.4, 100 mM NaCl, 1% NP40, 0.1% SDS, 0.5% sodium deoxycholate) with protease inhibitor (04693132001, Roche) and RNase inhibitor (Invitrogen). The lysate was precleared using Protein G Dynabeads (Thermo Scientific). BOLL antibody (5 μg) (13720‐1‐AP, Proteintech) was added to the precleared lysate and incubated with rotation at 4°C overnight. Protein G Dynabeads were resuspended using lysis buffer and added to the OLCs lysate and rotated at 4°C for 4 h. The lysate was put on a magnetic stand and the supernatant discarded. Beads were washed with lysis buffer for four times, rotated at 4°C for 5 min per wash. RNA was isolated using the TRIzol (15596026, Invitrogen) and the cDNA library was generated using the SMART‐Seq2 method followed by sequencing on the Illumina NovaSeq 6000 platform. Data analysis was performed following the published protocol [54]. Briefly, raw reads were trimmed to remove adaptor contaminants and low‐quality bases using Trimmomatic (version 0.36) with defined parameters. Sequencing adapter sequences were removed with Cutadapt‐4.1 (http://code.google.com/p/cutadapt/) and aligned to hg38 genome using HISAT 2.1.0. The Sam files were sorted and converted to Bam files by Samtools‐1.9. Reads were assembled and quantified by featureCounts (v1.6.5). Differential gene expression was calculated using an R package, edgeR and limma [55, 56]. Reads per kilobase per million were calculated from the normalisation results by the edgeR function reads per kilobase per million. To exclude potential false‐positive results, the RIP targets were defined as genes with a Log2(fold change) of ≥ 1 and *p* value < 0.05.

### 5.8 Transcriptome and Translatome Sequencing (T&T‐Seq) and Bioinformatics Analysis

The OLCs were processed using the published microscale T&T‐seq method [22] for library construction and sequencing analysis. Briefly, 100,000 OLCs lysates were partitioned into transcriptome (2 μL) and translatome (8 μL) fractions. Ribosome‐bound mRNAs were captured using RiboLace beads (Immagina) through 70‐min rotational incubation at 4°C. Post‐capture beads underwent two washes with W‐buffer (Immagina), followed by ribosome‐mRNA complex dissociation using 1% SDS and proteinase K treatment (37°C, 30 min). RNA was purified via TRIzol/chloroform extraction and precipitated overnight.

Full‐length cDNA synthesis was performed using the Single Cell Full‐Length mRNA‐Amplification Kit (Vazyme). Libraries were constructed with the TruePrep DNA Library Prep Kit V2 (Vazyme) and sequenced on the NovaSeq 6000 platform (Illumina) to achieve > 20 million 150‐bp paired‐end reads per sample.

Raw sequencing reads were subjected to quality control and adapter trimming using Trim Galore (v0.6.4) with the following parameters: trim_galore ‐q 20 ‐‐phred33 ‐‐stringency 3 ‐j 8 ‐‐length 50 ‐e 0.1 ‐‐paired XX_1.fq.gz XX_2.fq.gz ‐‐gzip ‐o output_path/. Processed reads were then aligned to the human reference genome (GRCh38/hg38, Gencode release v42) using HISAT2 (v2.1.0) [57] with the command: hisat2 ‐p 8 ‐x /path/to/genomeindex ‐1 XX_1.fq.gz −2 XX_2.fq.gz ‐S XX.sam ‐‐summary‐file XX.report. Gene annotation files (GTF format) were obtained from the Gencode database. Read quantification was performed using featureCounts (v1.6.5) [58] using the following parameters: featureCounts ‐T 32 ‐O ‐t exon ‐M ‐a /path/to/gtf ‐‐extraAttributes gene_name ‐o count.txt ‐p XX.sam. Transcript abundance was normalised as transcripts per million (TPM) using an in‐house Python script. For differential expression analysis of T&T‐seq data, DESeq2 [59] was implemented with default parameters. TE was calculated as TE = (1 + TPMtranslation)/(1 + TPMtranscription). Gene ontology (GO) and PPIs were performed using the Metascape website [60]. Default parameters were used. For GO enrichment, Min Overlap = 3, P value cutoff = 0.01, Min Enrichment = 1.5. For PPI, min network size = 3, max network size = 500 and physical core. The statistical significance of differentially expressed genes in KEGG database was detected with KOBAS v3.0 software. In the differential expression analysis, *p* < 0.05 and (FoldChange) ≥ 1.5 were the thresholds for significantly different expressions. Heatmap was generated by TBTools (v1.09854).

### 5.9 Dual‐Luciferase Reporter Assay

The 3′UTRs of human SYCP3, SYCE2, CENPL and LHX8, according to the sequences downloaded from the NCBI website, were cloned into the psiCHECK2 vector (Promega, C8021). One day before transfection, 293FT cells were transferred to a well in a 24‐well plate at a density of 1 × 104 cells per well. The next day, the 293FT cells were transfected in replicates of four with luciferase vectors carrying individual 3′UTRs and an overexpression p2k7 vector carrying BOLL or an empty vector using VigoFect (T001, VigoFect) in DPBS (20 ng dual‐luciferase vector and 160 ng overexpression vector). After 6 h of transfection, the transfection medium was replaced with 293FT medium and incubated at 37°C for 48 h. To measure the luciferase activity, the transfected cells were lysed and assayed using the Dual‐luciferase Reporter Assay System according to the manufacturer's protocol (DD1205, Vazyme). The relative luciferase activity was calculated by first normalising the values to the firefly/Renilla luciferase in the cells transfected with psiCheck2 empty vector followed by normalisation to the cells transfected with the empty overexpression vector.

### 5.10 In‐Gel Trypsin Digestion

The gel band of interest was excised from the gel, reduced with 5 mM of DTT and alkylated with 11 mM iodoacetamide which was followed by in‐gel digestion with sequencing grade modified trypsin at 37°C overnight. The peptides were extracted twice with 0.1% trifluoroacetic acid in 50% acetonitrile aqueous solution for 30 min. The peptide extracts were then centrifuged in a SpeedVac to reduce the volume.

### 5.11 Liquid Chromatography/Mass Spectrometry (LC/MS)/MS Analysis

The BOLL‐interacting proteome was obtained from cultured OLCs by IP, followed by boiling with SDS loading buffer and subjecting to preparative SDS‐PAGE. The proteins were subsequently visualised by Coomassie staining and the protein‐containing band was excised from the gel for LC/MS. LC/MS was performed at the Mass Spectrum Laboratory of Tsinghua University (China) with a Thermo‐Dionex Ultimate 3000 HPLC system (Thermo Scientific). The digestion products were separated by a 120 min gradient elution at a flow rate 0.300 μL/min with a Thermo‐Dionex Ultimate 3000 HPLC system which was directly interfaced with the Thermo Scientific Orbitrap Exploris 480 mass spectrometer. The analytical column was a home‐made fused silica capillary column (100 μm ID, 250 mm length; Upchurch, Oak Harbour, WA) packed with C‐18 resin (120 Å, 1.9 μm, Dr.Maisch, Ammerbuch, Germany). Mobile phase A consisted of 0.1% formic acid and mobile phase B consisted of 80% acetonitrile and 0.1% formic acid. The mass spectrometer was operated in the data‐dependent acquisition mode using Xcalibur 4.1 software and there is a single full‐scan mass spectrum in the Orbitrap (300–1600 m/z, 60,000) resolution followed by top‐speed MS/MS scans in the Orbitrap.

The MS/MS spectra from each LC–MS/MS run were searched against the *Homo sapiens* database downloaded from uniprot using an in‐house Proteome Discoverer (Version PD2.5, Thermo‐Fisher Scientific, USA). The search criteria were as follows: full tryptic specificity was required, two missed cleavage sites were allowed, Oxidation (M) was set as variable modification, Carbamidomethyl (C) was set as fixed modification, precursor ion mass tolerances were set at 20 ppm for all MS acquired in an Orbitrap mass analyser and the fragment ion mass tolerance was set at 0.02 Da for all MS2 spectra acquired. The peptide false discovery rate (FDR) was calculated using percolator provided by PD. FDR was determined based on PSMs when searched against the reverse, decoy database. Peptides only assigned to a given protein group were considered as unique. The FDR was also set to 0.01 for protein identifications.

### 5.12 Protein–Protein Interactions Network Analysis and Gene Function Analysis

Protein–protein interaction networks were conducted using the STRING database (v12.0), filtered at a combined confidence score threshold (> 0.4) to retain medium‐evidence interactions. All lines incorporated multi‐evidence integration scores derived from experimental, phylogenetic and computational predictions [61]. Networks were initially visualised using the STRINGdb R package plot_network function [62], followed by topology refinement in Cytoscape (v3.8.2) employing force‐directed layouts to resolve modular gene clusters. GO term annotations were systematically mapped to all nodes through database cross‐referencing.

### 5.13 Western Blotting

The protocol was performed as previously described [51, 63]. Briefly, proteins were isolated from cells by using RIPA lysis buffer and separated by 10% SDS‐polyacrylamide gel electrophoresis. Then they were transferred to PVDF membranes (ISEQ00010, Milipore). Thereafter, the membranes were incubated with primary antibodies against β‐Actin (sc‐47778, Santa Cruz Biotechnology), Anti‐alpha Tubulin (ab7291, Abcam), anti‐DYKDDDDK‐tag (M20008, Abmart), Mouse anti‐DAZL (MCA2336, Bio‐Rad), BOULE Polyclonal antibody (13720‐1‐AP, Proteintech), Rabbit Polyclonal SYCP3 Antibody (NB300‐232, Novus), FXR1 Polyclonal Antibody (13194‐1‐AP, Proteintech), PABP Polyclonal Antibody (ab21060, Abcam) at 4°C overnight and the secondary antibodies against rabbit (sc‐2357, Santa Cruz Biotechnology) and mouse (sc‐516102, Santa Cruz Biotechnology) were incubated for 1 h at room temperature. Immobilon Western kit (P90719, Millipore) was used to visualise the protein bands.

### 5.14 Semi‐Denaturing Detergent Agarose Gel Electrophoresis (SDD‐AGE) Assay

SDD‐AGE was adapted from previous protocols described by Halfmann et al., with minor modifications [64, 65]. BOLL full‐length proteins or deletion proteins were harvested and lysed in lysis buffer (50 mmol/L Tris–HCl, pH 7.5, 150 mmol/L NaCl, 0.5% Triton X‐100, 10% glycerol with 1× protease inhibitor cocktail for 30 min). Lysates were clarified twice through centrifugation at 14,000 rpm for 10 min at 4°C. 4× Sample loading buffer (2× TAE, 20% glycerol, 8% SDS, bromophenol blue) was added to lysates which were incubated for 10 min at room temperature, followed by loading with samples on newly prepared 1.5% agarose gel with 0.1% SDS and electrophoresis in running buffer (1× TAE and 0.1% SDS) for 4 h with a constant voltage of 30 V at 4°C. Proteins were then transferred to the PVDF membrane for Western blotting with indicated antibodies by capillary action.

### 5.15 Fluorescence Recovery After Photobleaching Analysis

BOLL‐mGFP‐FL proteins were used to label liquid droplets in vitro. We performed FRAP assaying in 293FT cells and FRAP experiments were performed on a confocal microscope (Ti‐2, Nikon) at room temperature. Defined regions were photobleached at 488 nm and the fluorescence intensities in these regions were collected every 1 s for in vitro droplets and normalised to the initial intensity before bleaching. Image intensity was measured in the region of interest.

### 5.16 Statistical Analysis

All Data were reported as mean ± standard deviation (SD) of at least three independent experiments (*n* ≥ 3). The data were analysed by a two‐tailed Student's *t*‐test. Differences were considered to be significant when *p* value was less than 0.05.

## Author Contributions

K.K. and Y.L. conceived the study and designed all experiments. Y.L. generated the expression vectors and performed most experiments with technical assistance by B.L. The RNA‐seq data was analysed by Y.L. The luciferase assay was performed by L.M. B.L. conducted the SDD‐AGE experiment of BOLL truncations. Protein‐RNA interaction prediction was performed by L.X. W.X. collected foetal human ovary samples. All authors discussed the results and Y.L. wrote the manuscript with comments from all authors.

## Funding

This work was supported by the National Natural Science Foundation of China (32350710190) and Ministry of Science and Technology of the People's Republic of China (2022YFA0806301 and 2021YFA0719301).

## Ethics Statement

All experiments were performed according to the ethical guidelines of Tsinghua University.

## Conflicts of Interest

The authors declare no conflicts of interest.

## Supporting information


**Figure S1:** BOLL is specifically expressed in human oogenesis during meiosis prophase. (A, B) Comparative triple‐staining of DAZL (Dazl, red), BOLL (Boll, green) and SYCP3 (Sycp3, cyan) in human foetal ovary (A) versus mouse embryonic ovary (B). Scale bars: 100 μm (overview), 20 μm (magnified). Triangle denotes BOLL (Boll)^+^ DAZL (Dazl)^+^ SYCP3 (Sycp3)^+^ germ cell, arrowhead indicates BOLL^+^ SYCP3^+^ germ cell. Scale bar: 20 μm. (C, D) Analysis of DAZL (Dazl), BOLL (Boll) and SYCP3 (Sycp3) co‐expression during meiotic progression in the human (C) and mouse (D) foetal ovary. BOLL exhibits preferential co‐occurrence with SYCP3^+^ cells in human foetal germ cells (60% overlap; *n* = 3; ****p < 0.0001), significantly exceeding other co‐expression patterns. In contrast, no cells co‐expressing only Boll and Sycp3 were detected in mouse foetal germ cells. (E, F) Comparative analysis of DAZL (Dazl) and BOLL (Boll) protein expression patterns during meiotic prophase.
**Figure S2:** BOLL promotes hESC meiotic differentiation in vitro. (A) Schematic of BOLL‐driven differentiation protocol for hESC meiosis induction. (B) DNA content analysis of hESCs transduced with BOLL and empty vector at differentiated Day 6. (2N (purple), S‐phase (yellow), 4N (green); > 5 × 10^5^ cells/condition, *n* = 3 independent experiments). (C) qRT‐PCR validation of meiotic and pluripotent genes in 4N‐enriched BOLL‐overexpressing cells vs. vector control (Vector). Data represent mean ± SD (*n* = 3 biological replicates; **p < 0.01, ***p < 0.001, ****p < 0.0001, Student's *t*‐test). (D) Magnified images of the meiotic spreads and the immunofluorescent staining of SYCP3 (green) and γH2AX (fuchsia) in induced hESCs. Scale bar: 10 μm.
**Figure S3:** Protein interaction network of high TE BOLL‐RIP‐RNAs. Protein interaction network analysis of high TE BOLL‐RIP‐RNAs. The network was analysed using STRING database (version 12.0). Nodes represent proteins and lines represent interactions. Solid lines indicate direct physical interactions, while dashed lines indicate indirect interactions.
**Figure S4:** AlphaFold3‐predicted analysis of binding sites and affinity between BOLL and target mRNAs. (A) Predicted interaction interface between full‐length BOLL (BOLL_FL, orange) and the full‐length SYCP3 3′UTR (SYCP3_3′UTR_FL, blue). The interaction is confined to the RRM domain of BOLL and a U‐rich region within the SYCP3 3′UTR. The black solid box outlines the interaction area. The inset shows a 90° rotated view of this region, highlighting putative residue‐level contacts (blue dotted lines), which represent *π*–*π* stacking interactions. (B) Predicted interaction interface between the RRM domain of BOLL (BOLL_RRM, orange) and the LHX8 binding sites (blue). The black solid box shows a magnified view of the binding site, detailing the interaction regions between the BOLL RRM and the four binding sites within LHX8. Blue and magenta dashed lines indicate predicted *π*–*π* stacking and salt bridge interactions, respectively. Structural predictions were generated using PyMOL v2.5. (C) Predicted binding free energy change (Δ*G*) for BOLL binding to target 3′UTRs and mutants. Δ*G* < 0 indicates spontaneity, more negative Δ*G* suggests higher complex stability. (D) Predicted dissociation constant (*K*_d_) for BOLL binding to target 3′UTRs and mutants. The *K*_d_ value represents the half‐saturation concentration; a lower *K*_d_ indicates a stronger predicted binding affinity. Prediction of Δ*G* and *K*_d_ was performed with PRA‐Pred [67].
**Figure S5:** Identification, network analysis and functional validation of BOLL‐interacting proteins. (A) Venn diagram depicting proteins significantly enriched in BOLL‐IP compared to IgG controls. The outer circles show 1174 validated proteins identified by MS in both IgG‐IP and BOLL‐IP groups, with criteria of a protein score ≥ 5, detection of ≥ 2 peptides and presence in both biological replicates. The inner circle highlights 125 proteins significantly enriched in BOLL‐IP relative to IgG‐IP (Fold change (BOLL‐IP/IgG) > 2, *p* < 0.05). (B) Protein–protein interaction network of representative BOLL‐IP proteins analysed via the STRING database. Nodes denote proteins, with solid lines indicating experimentally verified interactions and dashed lines representing predicted interactions. (C) Co‐IP assay of BOLL‐PABPC1 and BOLL‐FXR1 interaction in OLCs.
**Figure S6:** Punctate aggregation of BOLL in human foetal ovarian tissue and 293FT cells. (A) IF staining of human foetal ovary using anti‐BOLL (green) and anti‐SYCP3 (red) antibodies. White arrowheads indicate punctate BOLL signals. Scale bar, 20 μm. (B) Analysis of full‐length and truncated (Del‐3) BOLL proteins in 293FT cells by SDS‐PAGE and SDD‐AGE. Protein expression levels were normalised using anti‐Tubulin and anti‐FLAG antibodies on SDS‐PAGE gels (left). The presence of BOLL aggregates was confirmed by SDD‐AGE analysis (right) with an anti‐FLAG antibody. (C) FRAP analysis of BOLL‐mGFP in transfected 293FT cells. Representative images show a BOLL‐mGFP droplet before photobleaching (−4 s), immediately after bleaching (0 s) and following recovery (214 s). The white triangle marks the photobleached site. Scale bar, 20 μm.
**Figure S7:** Deletion of short segments of approximately 30 amino acids affects the formation of BOLL aggregates. (A) Diagram of BOLL deletion fragments (Del‐a to Del‐d). The blue line is the intrinsically disordered regions (IDRs) identified via MobiDB. (B) p2k7‐BOLL deletion plasmids (Del‐3, Del‐a to Del‐d) and the p2k7‐BOLL‐FL plasmid were packaged into lentivirus and used to infect differentiated hESCs. p2k7‐vector alone served as a negative control. After 6 days, cells were harvested for SDD‐AGE and SDS‐PAGE assays to detect BOLL aggregates and monomers using anti‐FLAG and anti‐α‐tubulin antibodies. (C) Schematic of BOLL deletion fragments (Del‐e to Del‐g). (D) p2k7‐BOLL deletion plasmids (Del‐4, Del‐e to Del‐g) were packaged into lentivirus and used to infect differentiated hESCs.
**Figure S8:** Protein–protein interaction network analysis of BOLL aggregates and Del‐3 enriched proteins. (A, B) Protein–protein interaction network analysis of full‐length BOLL protein (A) and the truncated Del‐3 protein (B) using the STRING database. The colour and lines coding of interaction types is consistent with Figure S5.
**Figure S9:** Formation of BOLL protein aggregates is independent of microfilament and microtubule assembly. (A) Immunofluorescence staining of microtubules and microfilaments in OLCs. The left panel shows microtubule staining after Nocodazole (NOC) treatment and the right panel shows microfilament staining after Cytochalasin D (Cyto D) treatment. Scale bar: 20 μm. (B) SDS‐PAGE and SDD‐AGE analysis of BOLL protein in OLCs following microfilament/microtubule inhibitor treatment.
**Figure S10:** AlphaFold3‐predicted structural models of BOLL and Del‐3 containing complexes. (A) Predicted interaction interface between BOLL (gold), FXR1 (purple) and PABPC1 (green). Interactions among protein residues near the DAZ domain are relatively clear, but the overall structure confidence is low due to the presence of low‐confidence disordered regions in all three proteins. The blue dashed line indicates the interaction area of the DAZ functional domain of the BOLL protein. The inset shows a 180°‐rotated view of the boxed region, highlighting putative residue‐level contacts (yellow dashes) with key residues labelled: BOLL (red), FXR1 (blue), PABPC1 (green). (B) Truncated Del‐3 (gold) exhibits disrupted binding capacity with FXR1 (purple) and PABPC1 (green). The blue dashed box indicates the position of the Del‐3 protein within the complex, revealing loss of defined interaction interfaces compared to wild‐type BOLL. Structural predictions generated using PyMOL v2.5.

**Table S1:** Primary antibodies used in this study.

**Table S2:** DNA primers used in this study.

## Data Availability

The MS proteomics data have been deposited to the ProteomeXchange Consortium (http://proteomecentral.proteomexchange.org) via the PRIDE partner repository [66] with the dataset identifier PXD068137. The RIP‐seq and T&T‐seq data generated in this study have been deposited in the NCBI Gene Expression Omnibus (GEO) database under accession code GSE307299. All data supporting the findings of this study are provided in the text of this paper and related Supporting Information.
