# Supplementary material for: BOLL‐Containing Aggregates Mediate the Translational Regulation During Human Oogenesis
Source: Cell Prolif. 2026 Feb 25;59(4):e70181. doi: 10.1111/cpr.70181 (PMC13052113; doi:10.1111/cpr.70181)
Supplement: Supplementary file 1 — Figure S1: BOLL is specifically expressed in human oogenesis during meiosis prophase. (A, B) Comparative triple‐staining of DAZL (Dazl, red), BOLL (Boll, green) and SYCP3 (Sycp3, cyan) in human foetal ovary (A) versus mouse embryonic ovary (B). Scale bars: 100 μm (overview), 20 μm (magnified). Triangle denotes BOLL (Boll)+ DAZL (Dazl)+ SYCP3 (Sycp3)+ germ cell, arrowhead indicates BOLL+ SYCP3+ germ cell. Scale bar: 20 μm. (C, D) Analysis of DAZL (Dazl), BOLL (Boll) and SYCP3 (Sycp3) co‐expression during meiotic progression in the human (C) and mouse (D) foetal ovary. BOLL exhibits preferential co‐occurrence with SYCP3+ cells in human foetal germ cells (60% overlap; n = 3; ****p < 0.0001), significantly exceeding other co‐expression patterns. In contrast, no cells co‐expressing only Boll and Sycp3 were detected in mouse foetal germ cells. (E, F) Comparative analysis of DAZL (Dazl) and BOLL (Boll) protein expression patterns during meiotic prophase. Figure S2: BOLL promotes hESC meiotic differentiation in vitro. (A) Schematic of BOLL‐driven differentiation protocol for hESC meiosis induction. (B) DNA content analysis of hESCs transduced with BOLL and empty vector at differentiated Day 6. (2N (purple), S‐phase (yellow), 4N (green); > 5 × 10^5^ cells/condition, n = 3 independent experiments). (C) qRT‐PCR validation of meiotic and pluripotent genes in 4N‐enriched BOLL‐overexpressing cells vs. vector control (Vector). Data represent mean ± SD (n = 3 biological replicates; **p < 0.01, ***p < 0.001, ****p < 0.0001, Student's t‐test). (D) Magnified images of the meiotic spreads and the immunofluorescent staining of SYCP3 (green) and γH2AX (fuchsia) in induced hESCs. Scale bar: 10 μm. Figure S3: Protein interaction network of high TE BOLL‐RIP‐RNAs. Protein interaction network analysis of high TE BOLL‐RIP‐RNAs. The network was analysed using STRING database (version 12.0). Nodes represent proteins and lines represent interactions. Solid lines indicate direct physical interaction [file CPR-59-e70181-s001.docx]

**Supplementary Table 1. Primary antibodies used in this study.**

| Primary antibodies | Source | Dilution | Cat. no. |
| --- | --- | --- | --- |
| γH2AX | Mouse monoclonal | 1:100 | ab26350, Abcam |
| SYCP3 | Rabbit polyclonal | 1:100 | NB300-232, Novus |
| DAZL | Mouse monoclonal | IF, 1:50; Western 1:500 | MCA2336, AbD Serotec |
| BOLL | Rabbit polyclonal | IF, 1:200; Western 1:1000 | 13720-1-AP, Proteintech |
| α tubulin | Mouse monoclonal | Western 1:5000 | ab7291, Abcam |
| PABP | Rabbit polyclonal | IF, 1:200; Western 1:1000 | ab21060, Abcam |
| FXR1 | Rabbit polyclonal | IF, 1:100; Western 1:1000 | 13194-1-AP, Proteintech |
| β actin | Rabbit monoclonal | Western 1:5000 | AC048, Abclonal |
| Anti-DYKDDDDK-Tag | Mouse monoclonal | Western 1:1000 | M20008, Abmart |
