# Supplementary material for: BOLL‐Containing Aggregates Mediate the Translational Regulation During Human Oogenesis
Source: Cell Prolif. 2026 Feb 25;59(4):e70181. doi: 10.1111/cpr.70181 (PMC13052113; doi:10.1111/cpr.70181)
Supplement: Supplementary file 2 — Table S1: Primary antibodies used in this study. [file CPR-59-e70181-s002.docx]

**Supplementary Table 2. DNA Primers used in this study.**

| Gene | Forward Primer | Reverse Primer |
| --- | --- | --- |
| *SYCP3* | AAATCTGGGAAGCCGTCTGT | AACTCCAACTCCTTCCAGCA |
| *SYCP2* | ACCAGATTCACAGGCAGCGGAA | GGTGTCCAACATGCCCATTTGC |
| *GAPDH* | GAGTCAACGGATTTGGTCGT | TTGATTTTGGAGGGATCTCG |
| *LHX8* | CAAGCACAATTTGCTCAGGA | TGGCGTGCTCTACAATTCTG |
| *ACTB* | GGCATCCACGAAACTACCTT | CTCGTCATACTCCTGCTTGC |
| *NANOG* | TTCCTTCCTCCATGGATCTG | TCTGGAACCAGGTCTTCACC |
| *OCT4* | AGTGAGAGGCAACCTGGAGA | GTGAAGTGAGGGCTCCCATA |
| *ZGLP1* | CGCCTGTGGGATCAGGTACAAG | TCCAGGGACACTCCACATCT |
| *MEIOC* | GAGTGCTGTGAACAATGGAGAGC | GATGGGTTGGAAGTCAGCCTTG |
| *BOLL* | ATGTAGCTCCCCTGTGATGG | TGATGGCACTTGGAGCATAA |
| *STRA8* | CCTCAAAGTGGCAGGTTCTGAA | TCCTCTAAGCTGCTTGCATGC |
