# Supplementary material for: BOLL‐Containing Aggregates Mediate the Translational Regulation During Human Oogenesis
Source: Cell Prolif. 2026 Feb 25;59(4):e70181. doi: 10.1111/cpr.70181 (PMC13052113; doi:10.1111/cpr.70181)
Supplement: Supplementary file 3 — Table S2: DNA primers used in this study. [file CPR-59-e70181-s003.pdf]

Figure S1

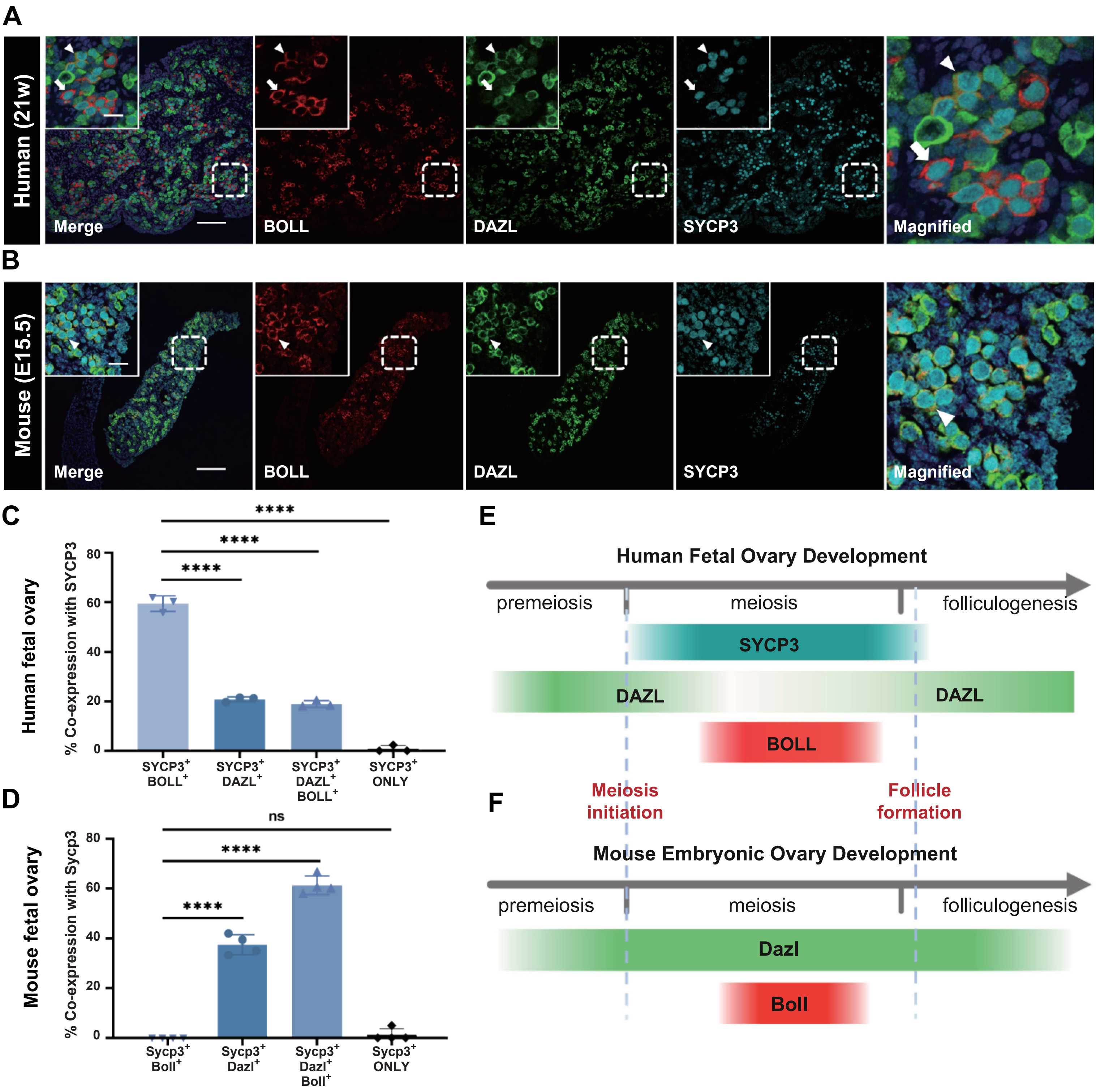

A
Human (21w)
Merge
BOLL
DAZL
SYCP3
Magnified
B
Mouse (E15.5)
Merge
BOLL
DAZL
SYCP3
Magnified
C
Human fetal ovary
% Co-expression with SYCP3
****
****
****
80
60
40
20
0
SYCP3+ BOLL+
SYCP3+ DAZL+
SYCP3+ DAZL+ BOLL+
SYCP3+ ONLY
D
Mouse fetal ovary
% Co-expression with Sycp3
ns
****
****
80
60
40
20
0
Sycp3+ Boll+
Sycp3+ Dazl+
Sycp3+ Dazl+ Boll+
Sycp3+ ONLY
E
Human Fetal Ovary Development
premeiosis
meiosis
folliculogenesis
SYCP3
DAZL
DAZL
BOLL
Meiosis initiation
Follicle formation
F
Mouse Embryonic Ovary Development
premeiosis
meiosis
folliculogenesis
Dazl
Boll

Figure S2

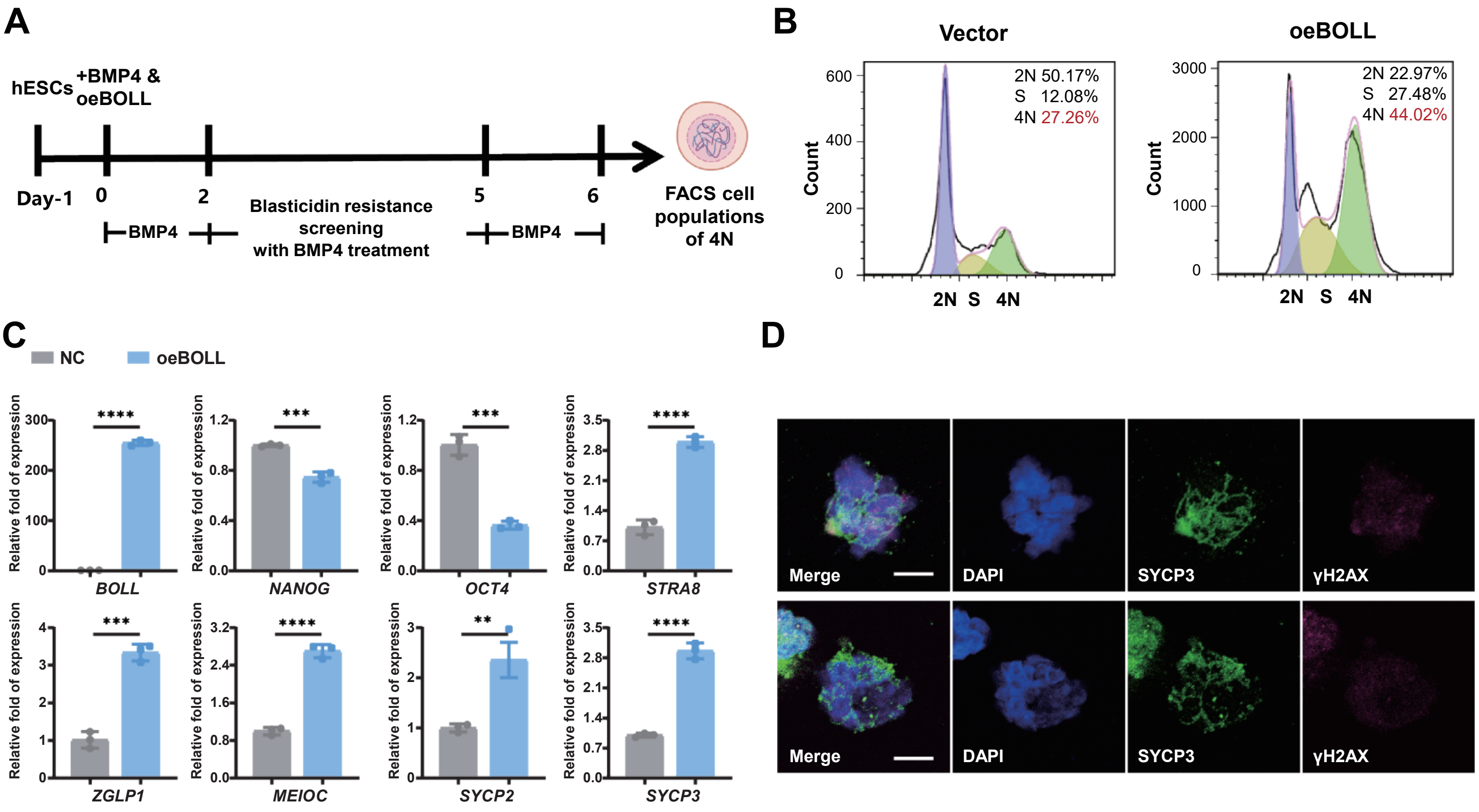

A
hESCs
+BMP4 &
oeBOLL
Day-1
0
2
5
6
BMP4
Blasticidin resistance
screening
with BMP4 treatment
BMP4
FACS cell
populations
of 4N
B
Vector
oeBOLL
Count
2N 50.17%
S 12.08%
4N 27.26%
2N 22.97%
S 27.48%
4N 44.02%
2N S 4N
C
NC
oeBOLL
Relative fold of expression
BOLL
NANOG
OCT4
STRA8
ZGLP1
MEIOC
SYCP2
SYCP3
D
Merge
DAPI
SYCP3
γH2AX

Figure S3

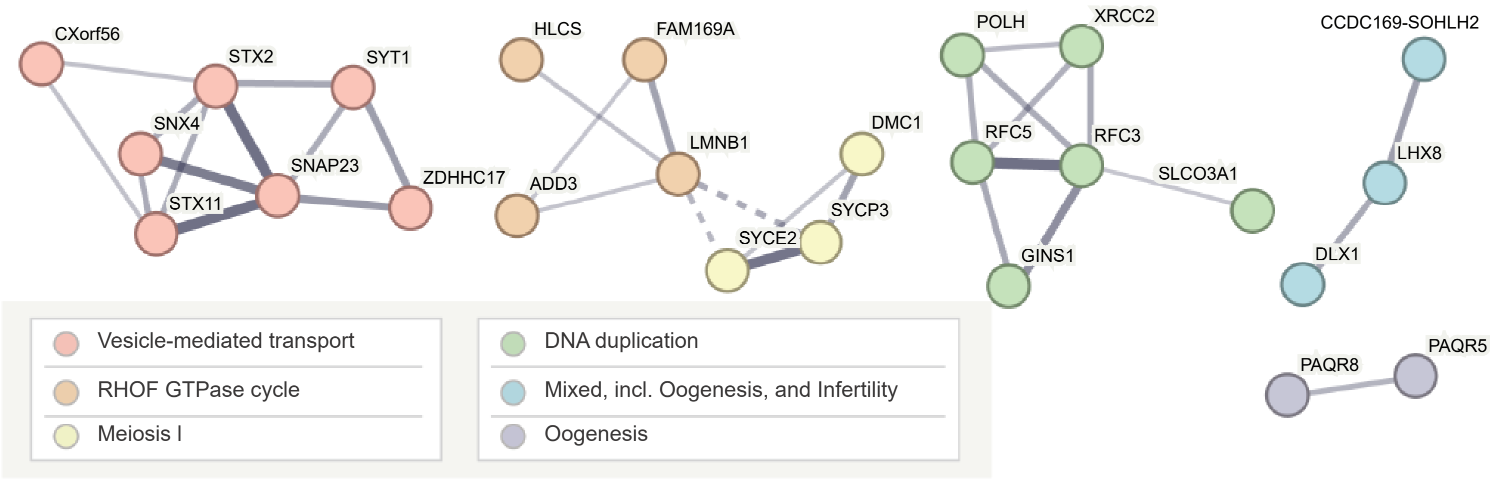
CXorf56
STX2
SYT1
SNX4
SNAP23
ZDHHC17
STX11
HLCS
FAM169A
LMNB1
ADD3
DMC1
SYCP3
SYCE2
POLH
XRCC2
RFC5
RFC3
SLCO3A1
GINS1
CCDC169-SOHLH2
LHX8
DLX1
PAQR8
PAQR5
Vesicle-mediated transport
RHOF GTPase cycle
Meiosis I
DNA duplication
Mixed, incl. Oogenesis, and Infertility
Oogenesis

Figure S4

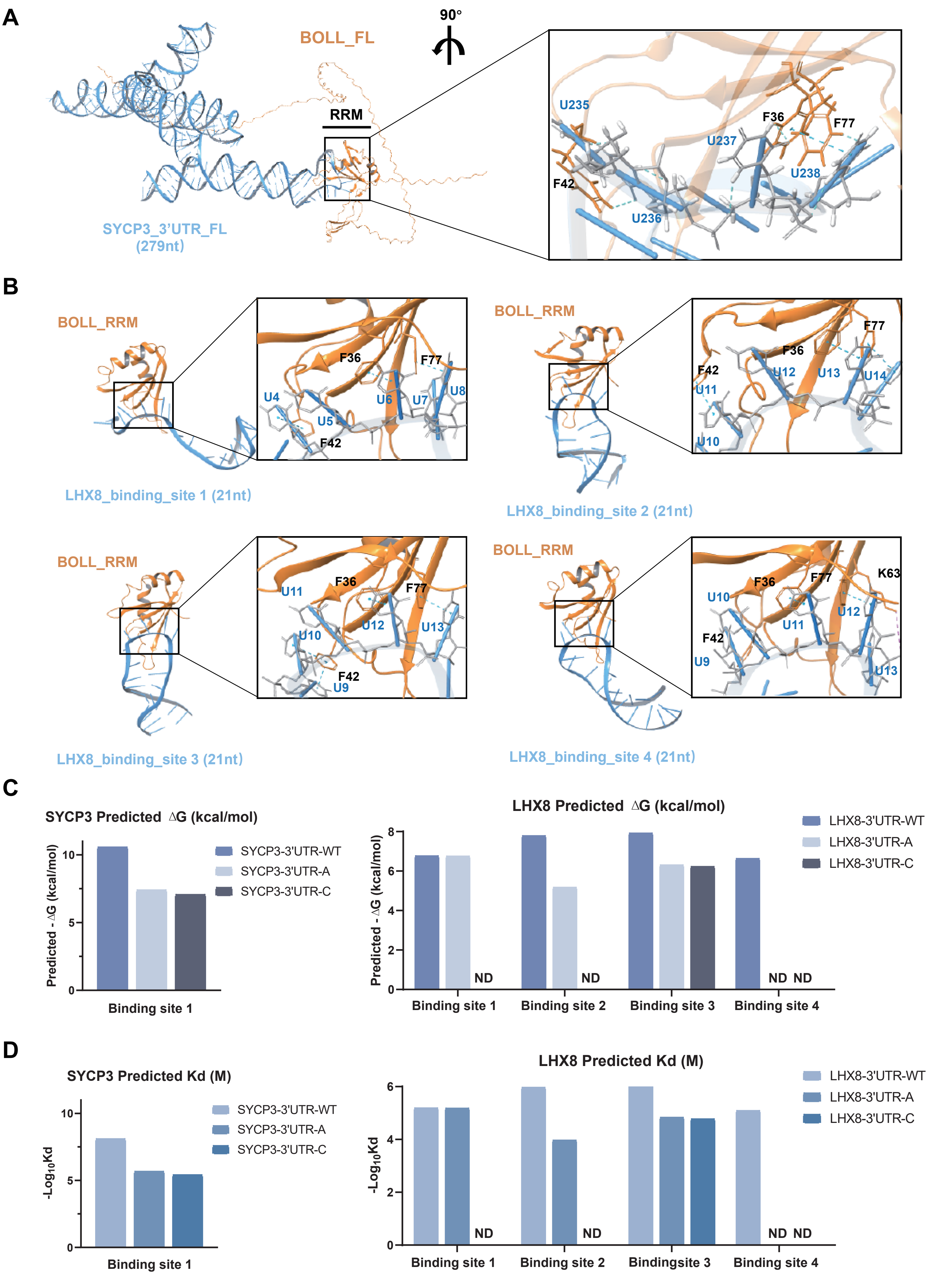
A
BOLL_FL
90°
RRM
SYCP3_3'UTR_FL
(279nt)
U235
F36
F77
U237
U238
F42
U236
B
BOLL_RRM
F36
F77
U4
U6
U7
U8
U5
F42
LHX8_binding_site 1 (21nt)
BOLL_RRM
F77
F36
F42
U12
U13
U14
U11
U10
LHX8_binding_site 2 (21nt)
BOLL_RRM
U11
F36
F77
U12
U13
U10
F42
U9
LHX8_binding_site 3 (21nt)
BOLL_RRM
K63
F36
F77
U10
U12
U11
F42
U9
U13
LHX8_binding_site 4 (21nt)
C
SYCP3 Predicted ΔG (kcal/mol)
Predicted - ΔG (kcal/mol)
SYCP3-3'UTR-WT
SYCP3-3'UTR-A
SYCP3-3'UTR-C
Binding site 1
LHX8 Predicted ΔG (kcal/mol)
Predicted - ΔG (kcal/mol)
LHX8-3'UTR-WT
LHX8-3'UTR-A
LHX8-3'UTR-C
ND
ND
ND ND
Binding site 1
Binding site 2
Binding site 3
Binding site 4
D
SYCP3 Predicted Kd (M)
$-Log_{10}Kd$
SYCP3-3'UTR-WT
SYCP3-3'UTR-A
SYCP3-3'UTR-C
Binding site 1
LHX8 Predicted Kd (M)
$-Log_{10}Kd$
LHX8-3'UTR-WT
LHX8-3'UTR-A
LHX8-3'UTR-C
ND
ND
ND ND
Binding site 1
Binding site 2
Bindingsite 3
Binding site 4

Figure S5

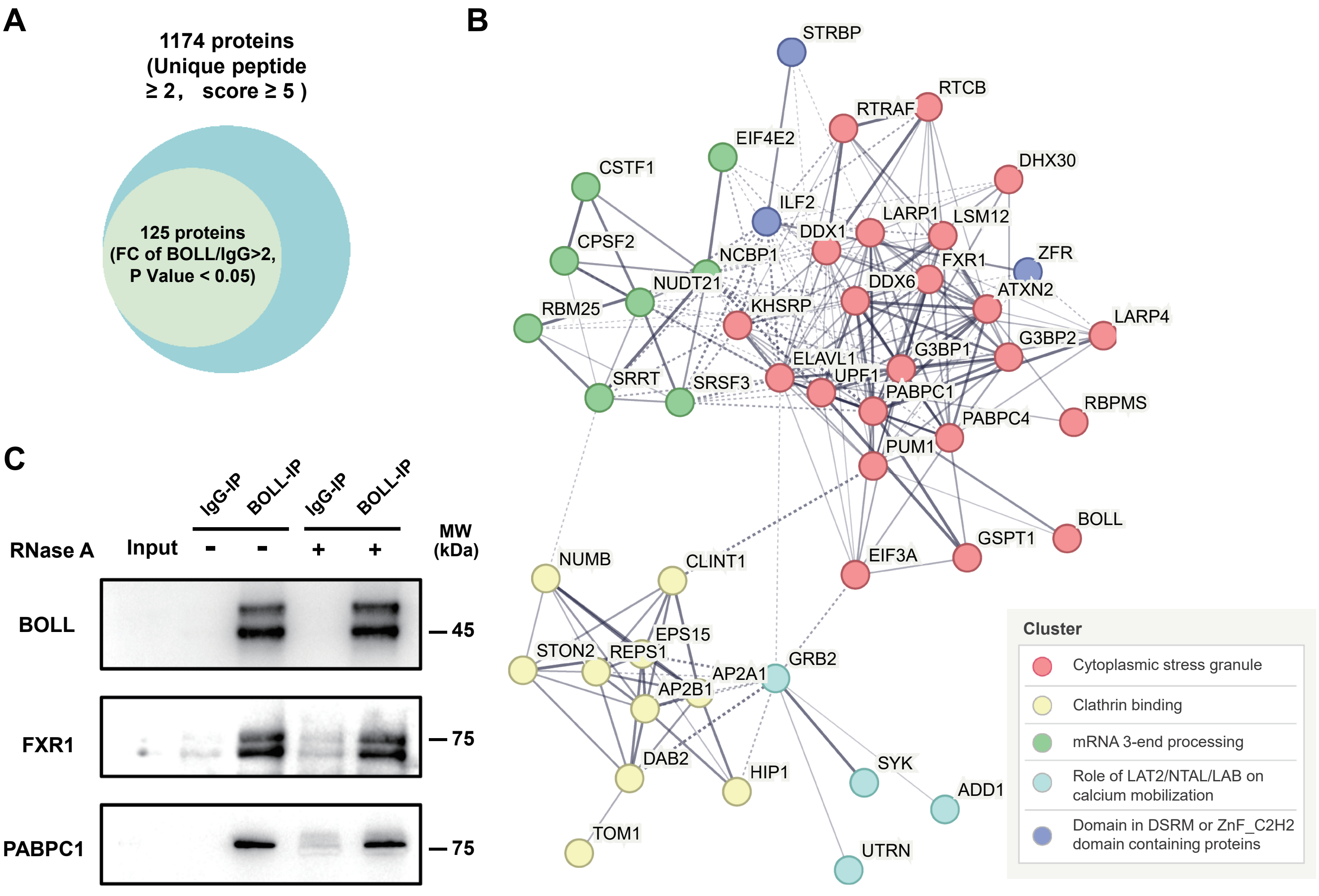
A
1174 proteins
(Unique peptide
≥ 2， score ≥ 5)
125 proteins
(FC of BOLL/IgG>2,
P Value < 0.05)
B
STRBP
RTCB
RTRAF
EIF4E2
DHX30
CSTF1
ILF2
LARP1
LSM12
DDX1
CPSF2
NCBP1
FXR1
ZFR
NUDT21
DDX6
ATXN2
RBM25
KHSRP
LARP4
G3BP2
G3BP1
ELAVL1
SRRT
UPF1
SRSF3
PABPC1
RBPMS
PABPC4
PUM1
BOLL
GSPT1
NUMB
CLINT1
EIF3A
EPS15
STON2
REPS1
GRB2
AP2A1
AP2B1
SYK
DAB2
HIP1
ADD1
TOM1
UTRN
Cluster
Cytoplasmic stress granule
Clathrin binding
mRNA 3-end processing
Role of LAT2/NTAL/LAB on calcium mobilization
Domain in DSRM or ZnF_C2H2 domain containing proteins
C
IgG-IP
BOLL-IP
IgG-IP
BOLL-IP
RNase A
Input
-
-
+
+
MW (kDa)
BOLL
45
FXR1
75
PABPC1
75

Figure S6

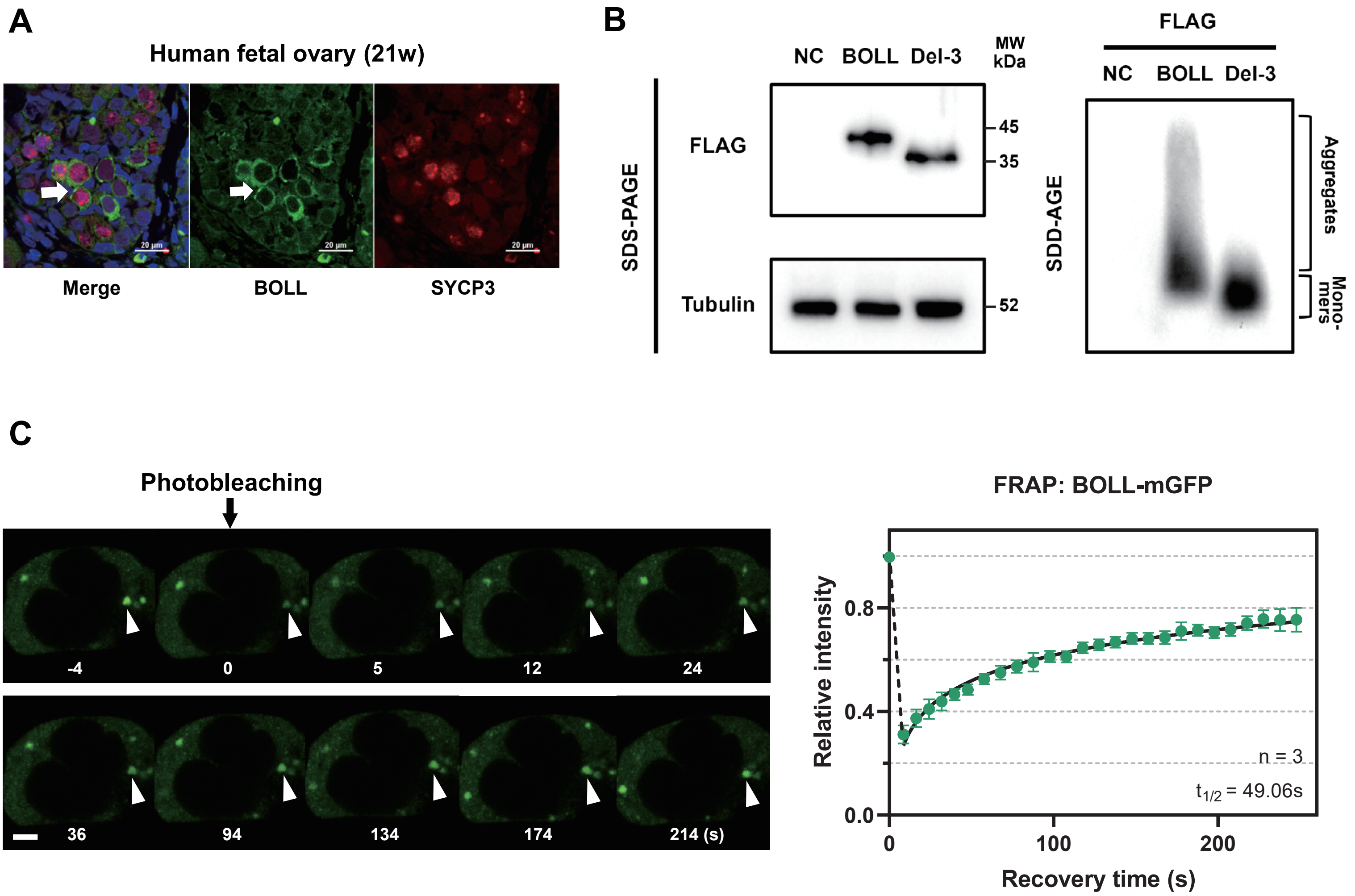
A
Human fetal ovary (21w)
20 μm
Merge
BOLL
SYCP3
B
NC
BOLL
Del-3
MW kDa
SDS-PAGE
FLAG
45
35
Tubulin
52
FLAG
SDD-AGE
Aggregates
Mono-mers
C
Photobleaching
-4
0
5
12
24
36
94
134
174
214 (s)
FRAP: BOLL-mGFP
Relative intensity
0.0
0.4
0.8
0
100
200
Recovery time (s)
n = 3
t1/2 = 49.06s

Figure S7

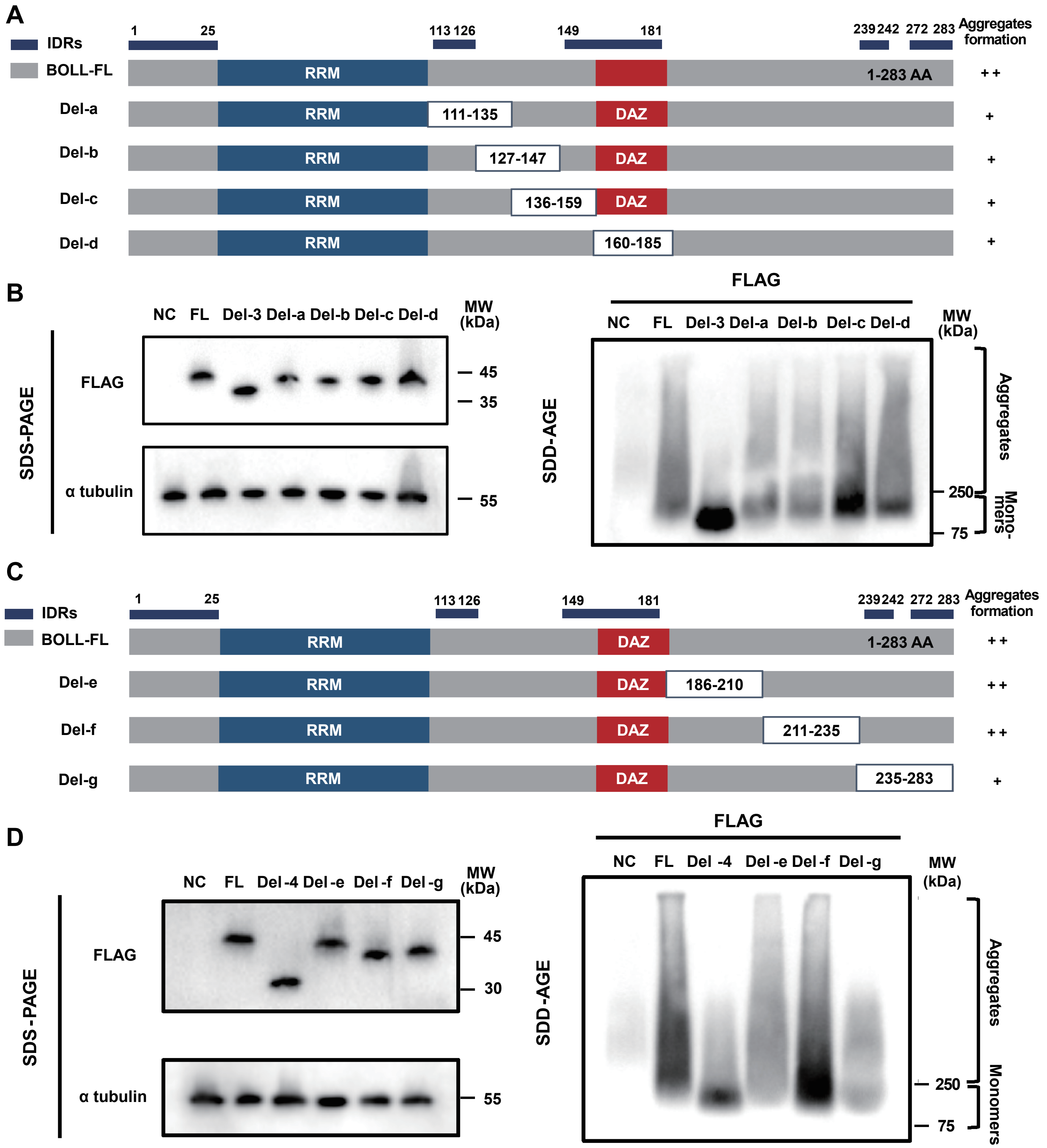

A
IDRs
BOLL-FL
1
25
113
126
149
181
239
242
272
283
Aggregates formation
RRM
1-283 AA
++
Del-a
111-135
DAZ
+
Del-b
127-147
Del-c
136-159
Del-d
160-185
B
NC
FL
Del-3
Del-a
Del-b
Del-c
Del-d
MW (kDa)
SDS-PAGE
FLAG
45
35
α tubulin
55
SDD-AGE
Aggregates
Mono-mers
250
75
C
Del-e
186-210
Del-f
211-235
Del-g
235-283
D
Del-4
30
Monomers

Figure S8

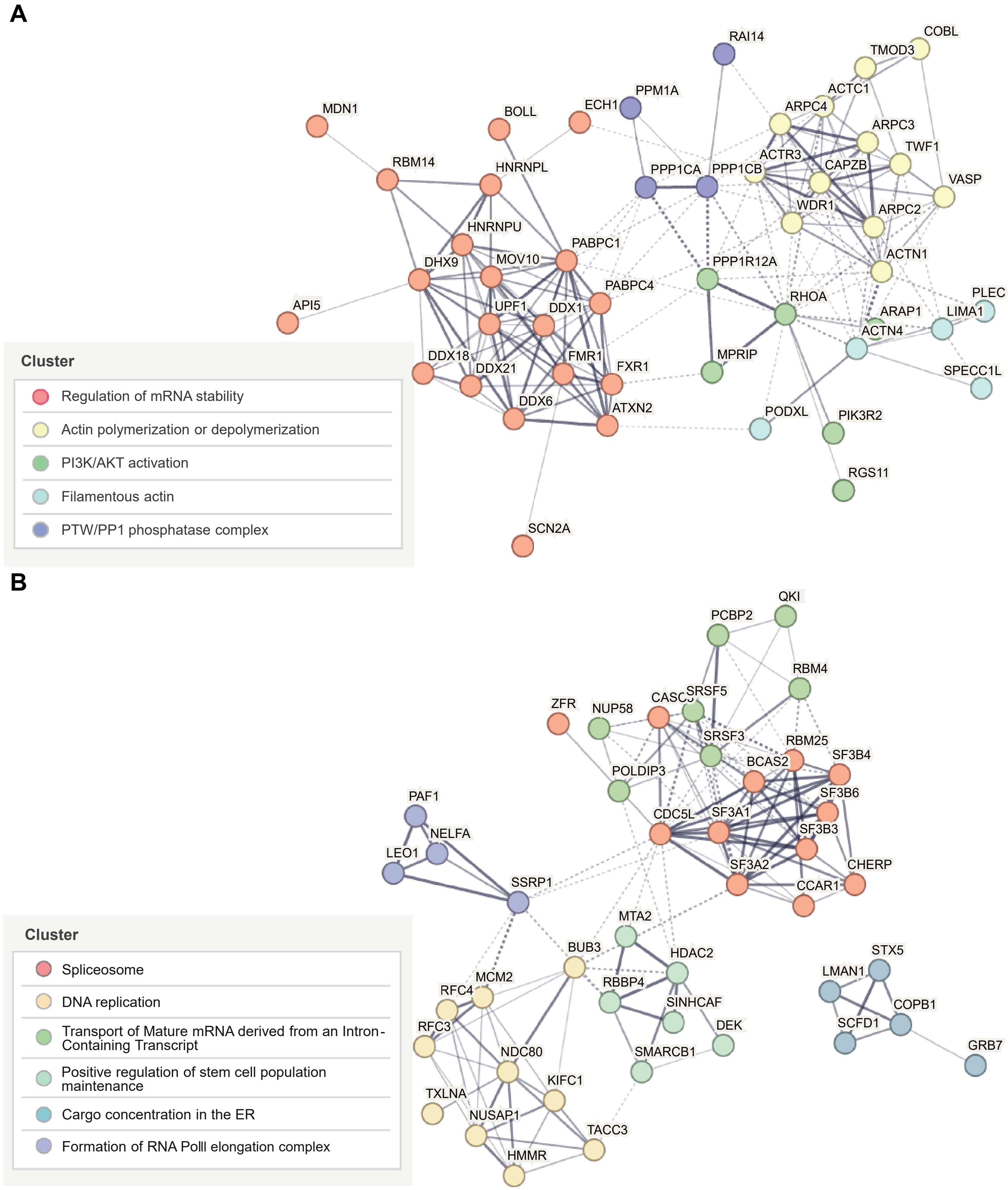

A
MDN1
BOLL
ECH1
PPM1A
RAI14
COBL
TMOD3
ACTC1
ARPC4
ARPC3
TWF1
RBM14
HNRNPL
PPP1CA
PPP1CB
ACTR3
CAPZB
VASP
WDR1
ARPC2
HNRNPU
PABPC1
ACTN1
DHX9
MOV10
PPP1R12A
PABPC4
RHOA
PLEC
API5
UPF1
DDX1
ARAP1
LIMA1
ACTN4
DDX18
FMR1
MPRIP
DDX21
FXR1
SPECC1L
DDX6
ATXN2
PODXL
PIK3R2
RGS11
SCN2A
Cluster
Regulation of mRNA stability
Actin polymerization or depolymerization
PI3K/AKT activation
Filamentous actin
PTW/PP1 phosphatase complex
B
QKI
PCBP2
RBM4
ZFR
NUP58
CASC3
SRSF5
SRSF3
RBM25
BCAS2
SF3B4
POLDIP3
SF3B6
PAF1
NELFA
CDC5L
SF3A1
SF3B3
LEO1
SF3A2
CHERP
SSRP1
CCAR1
MTA2
BUB3
HDAC2
STX5
MCM2
RBBP4
LMAN1
RFC4
SINHCAF
COPB1
RFC3
DEK
SCFD1
NDC80
SMARCB1
GRB7
KIFC1
TXLNA
NUSAP1
TACC3
HMMR
Cluster
Spliceosome
DNA replication
Transport of Mature mRNA derived from an Intron-Containing Transcript
Positive regulation of stem cell population maintenance
Cargo concentration in the ER
Formation of RNA PolII elongation complex

Figure S9

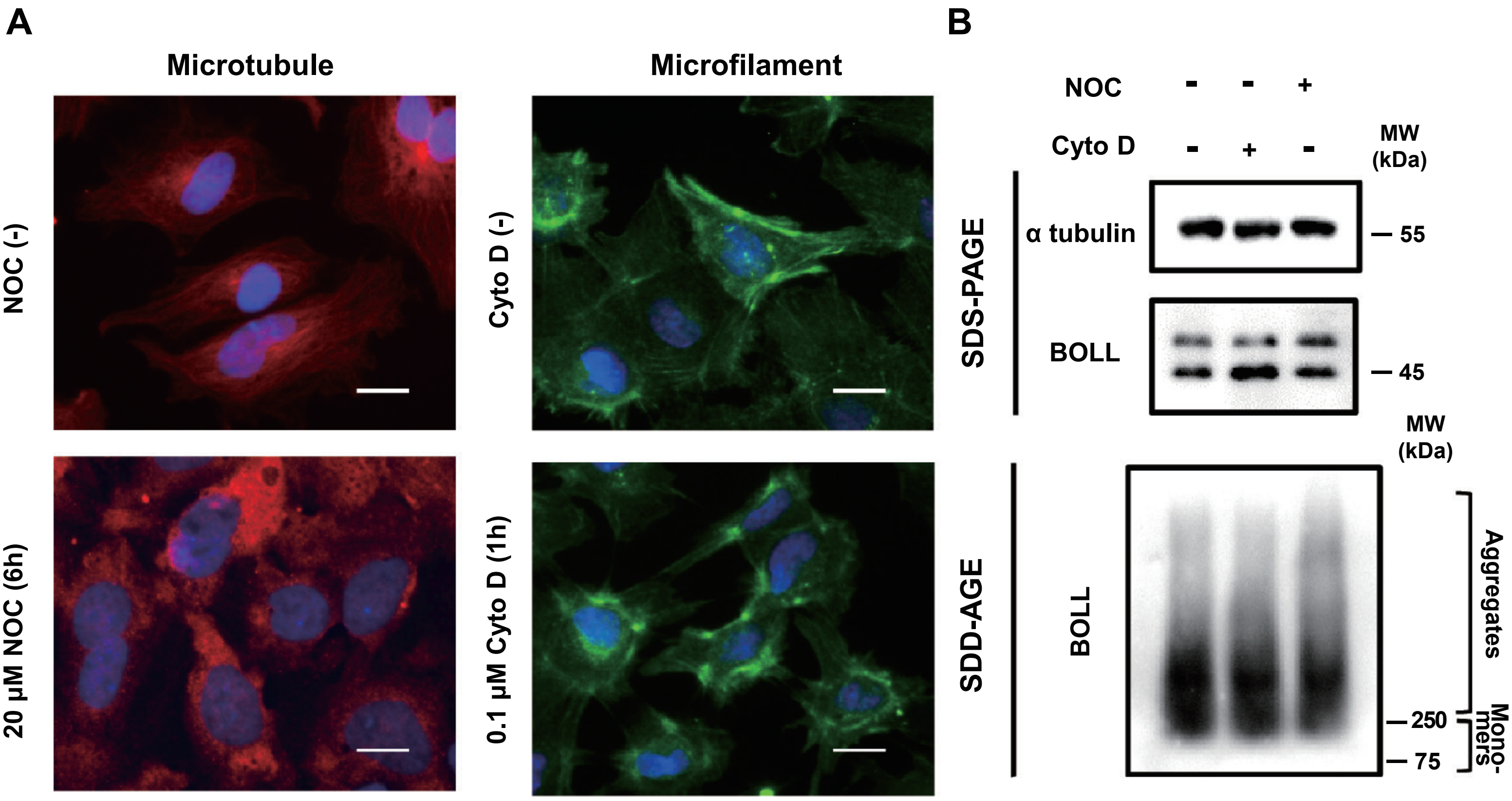
A
Microtubule
Microfilament
NOC (-)
20 µM NOC (6h)
Cyto D (-)
0.1 µM Cyto D (1h)
B
NOC - - +
Cyto D - + -
MW (kDa)
SDS-PAGE
α tubulin
55
BOLL
45
MW (kDa)
SDD-AGE
BOLL
Aggregates
Mono-mers
250
75

Figure S10

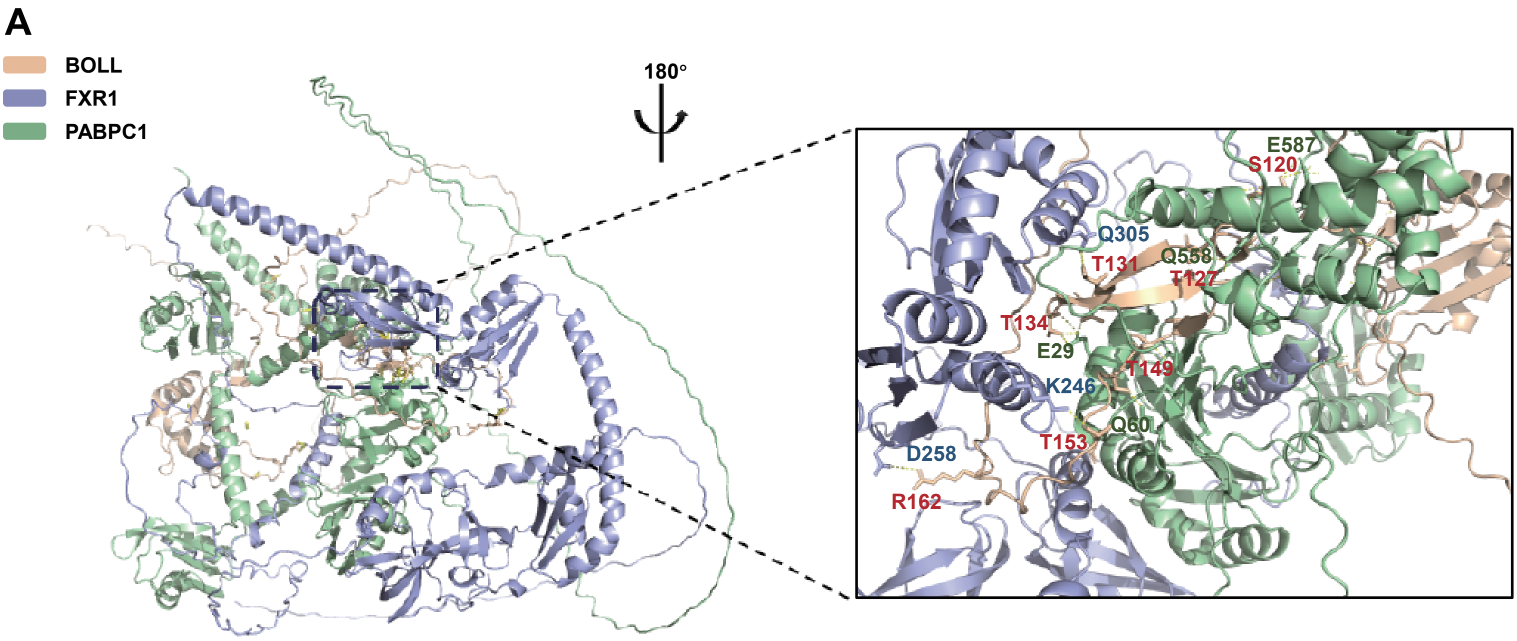
A
BOLL
FXR1
PABPC1
180°
E587
S120
Q305
T131
Q558
T127
T134
E29
T149
K246
Q60
D258
T153
R162

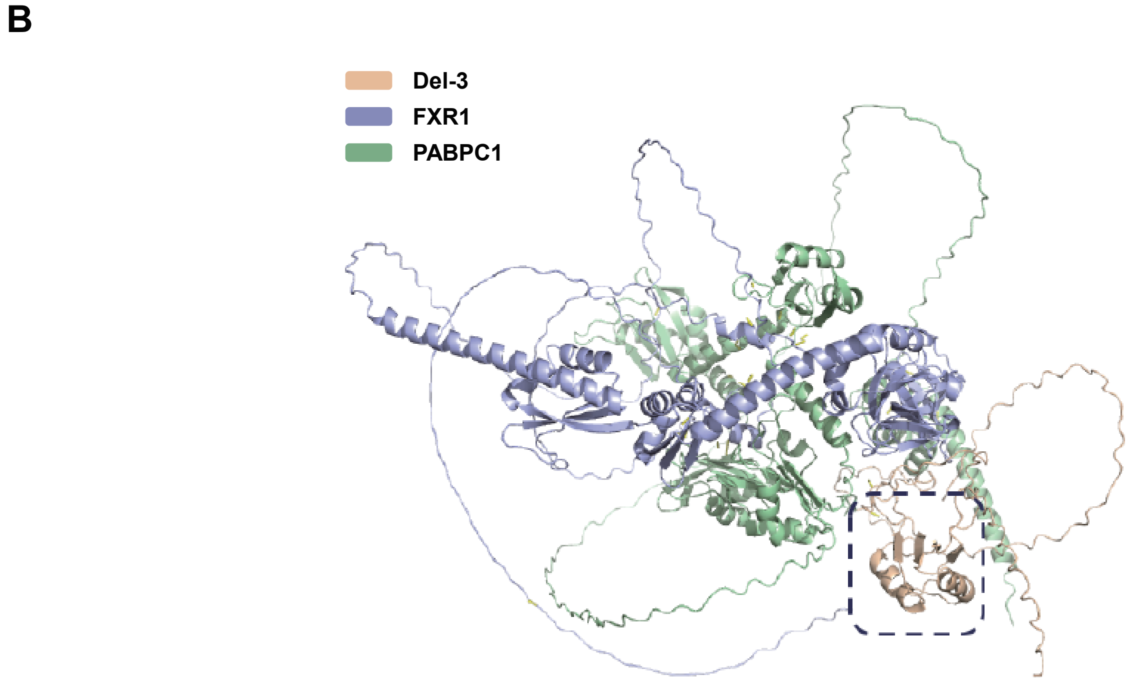
B
Del-3
FXR1
PABPC1
